# Glutathione Precursor *N*-Acetyl-Cysteine Modulates EEG Synchronization in Schizophrenia Patients: A Double-Blind, Randomized, Placebo-Controlled Trial

**DOI:** 10.1371/journal.pone.0029341

**Published:** 2012-02-22

**Authors:** Cristian Carmeli, Maria G. Knyazeva, Michel Cuénod, Kim Q. Do

**Affiliations:** 1 Center for Psychiatric Neuroscience, Department of Psychiatry, Centre Hospitalier Universitaire Vaudois (CHUV), University of Lausanne, Lausanne, Switzerland; 2 Laboratoire de Recherche en Neuroimagerie (LREN), Département des Neurosciences Cliniques (DNC), Centre Hospitalier Universitaire Vaudois (CHUV), University of Lausanne, Lausanne, Switzerland; 3 Department of Radiology, Centre Hospitalier Universitaire Vaudois (CHUV), University of Lausanne, Lausanne, Switzerland; University of Queensland, Australia

## Abstract

Glutathione (GSH) dysregulation at the gene, protein, and functional levels has been observed in schizophrenia patients. Together with disease-like anomalies in GSH deficit experimental models, it suggests that such redox dysregulation can play a critical role in altering neural connectivity and synchronization, and thus possibly causing schizophrenia symptoms. To determine whether increased GSH levels would modulate EEG synchronization, N-acetyl-cysteine (NAC), a glutathione precursor, was administered to patients in a randomized, double-blind, crossover protocol for 60 days, followed by placebo for another 60 days (or vice versa). We analyzed whole-head topography of the multivariate phase synchronization (MPS) for 128-channel resting-state EEGs that were recorded at the onset, at the point of crossover, and at the end of the protocol. In this proof of concept study, the treatment with NAC significantly increased MPS compared to placebo over the left parieto-temporal, the right temporal, and the bilateral prefrontal regions. These changes were robust both at the group and at the individual level. Although MPS increase was observed in the absence of clinical improvement at a group level, it correlated with individual change estimated by Liddle's disorganization scale. Therefore, significant changes in EEG synchronization induced by NAC administration may precede clinically detectable improvement, highlighting its possible utility as a biomarker of treatment efficacy.

**Trial Registration:**

ClinicalTrials.gov NCT01506765

## Introduction

Evidence increasingly points to the involvement of oxidative stress in schizophrenia pathophysiology [1]. In the last few years, we have shown that a redox dysregulation due to a glutathione (GSH) synthesis deficit of genetic origin represents one of the major risk factors for schizophrenia [2]. Indeed, patients suffering from schizophrenia manifest genetically determined deficits in the GSH system. Specifically, (i) GSH levels in the brain and cerebrospinal fluid of patients are decreased [3]–[5]; (ii) under oxidative stress conditions, activity of the key GSH synthesizing enzyme, glutamate cysteine ligase (GCL), and the GSH levels in patients' fibroblasts are diminished [6], and (iii) allelic variants of the GCL modulatory subunit (*gclm*) [7] and catalytic subunit (*gclc*) [6] genes are associated with the disease.

In particular, in two case-control studies, a GAG trinucleotide with 7, 8, or 9 repeats polymorphism in the *gclc* gene showed an association with schizophrenia [6]. This polymorphism has functional consequences: subjects with the ‘high risk’ genotypes (8/7, 8/8, 8/9, and 9/9) showed lower GCL activity, *gclc* protein expression, and GSH content than subjects with ‘low risk’ genotypes (7/7 and 7/9). Moreover, in pharmacological and knock-out models, developmental GSH deficit induces morphological, neurophysiological, and behavioral anomalies analogous to those reported in schizophrenia patients [8]–[15].

A developmental dysregulation of GSH synthesis of genetic origin, when combined with environmental risk factors, which generate oxidative stress at specific developmental stages, may affect functional connectivity – in particular, neural synchronization ([2], see also the Discussion). This is likely mediated by hypoactive N-methyl-D-aspartate (NMDA) receptors, impairment of parvalbumin immunoreactive fast-spiking GABA interneurons, imbalance between excitatory and inhibitory activity in temporal and prefrontal cortices, and damage to myelination, resulting in altered local oscillations and long-range neural synchronization [2].

The fact that schizophrenia is associated with abnormalities in neural synchrony is well documented. Abnormal oscillations and/or distance synchronization are manifested in the resting state [16]–[20], perceptual grouping [21], attention [22], [23], working memory [24]–[26], consciousness [27], and other cognitive/behavioral responses (for review see [28]). Therefore, the abnormalities of synchronization might represent a core pathophysiological mechanism for psychotic and cognitive disturbances.

In a recent double-blind, randomized, placebo-controlled multicenter clinical trial, the supplementation of N-acetyl-cysteine (NAC), a GSH precursor and an antioxidant, led to the improvement of the negative symptoms and reduced side effects of antipsychotics in a cohort of 140 schizophrenia patients [29]. Based on this promising result and on the assumption that abnormal neural synchronization in schizophrenia is related to redox dysregulation/oxidative stress, we proposed that NAC may have a potential effect on EEG synchronization. To support this hypothesis, we examine in the present proof of concept study the effects of NAC treatment on the whole-head topography of multivariate EEG synchronization in a small subgroup of schizophrenia patients from the Swiss sample of the above described NAC clinical trial.

## Materials and Methods

### Clinical Trial Protocol

The supporting CONSORT checklist is available as supporting information; see Checklist S1. NAC (2 g daily) and placebo were administered to schizophrenia patients in a double-blind crossover design. The clinical trial was conducted from November 2003 to November 2005. One group received NAC for the first 2 months and then placebo for another period of 2 months, and the other group received placebo first and then NAC. NAC was purchased from Zambon (Italy). NAC and placebo capsules were manufactured by DFC Thompson (Sydney, Australia) and re-conditioned by a pharmacist of the Department of Psychiatry of the Centre Hospitalier Universitaire Vaudois and University of Lausanne. EEG recordings were performed at the onset of the protocol (baseline measurements), at the point of crossover, and at the end of the study. In keeping with the main trial protocol [29], psychopathological scales including the Positive and Negative Symptoms Scale (PANSS) were evaluated every 2 weeks. The three syndromes including a negative, a positive, and a disorganization factor have been computed according to Liddle's model [30]. A copy of the trial protocol is included in the Supporting Information; see Protocol S1.

#### Participants

**Figure 1:**
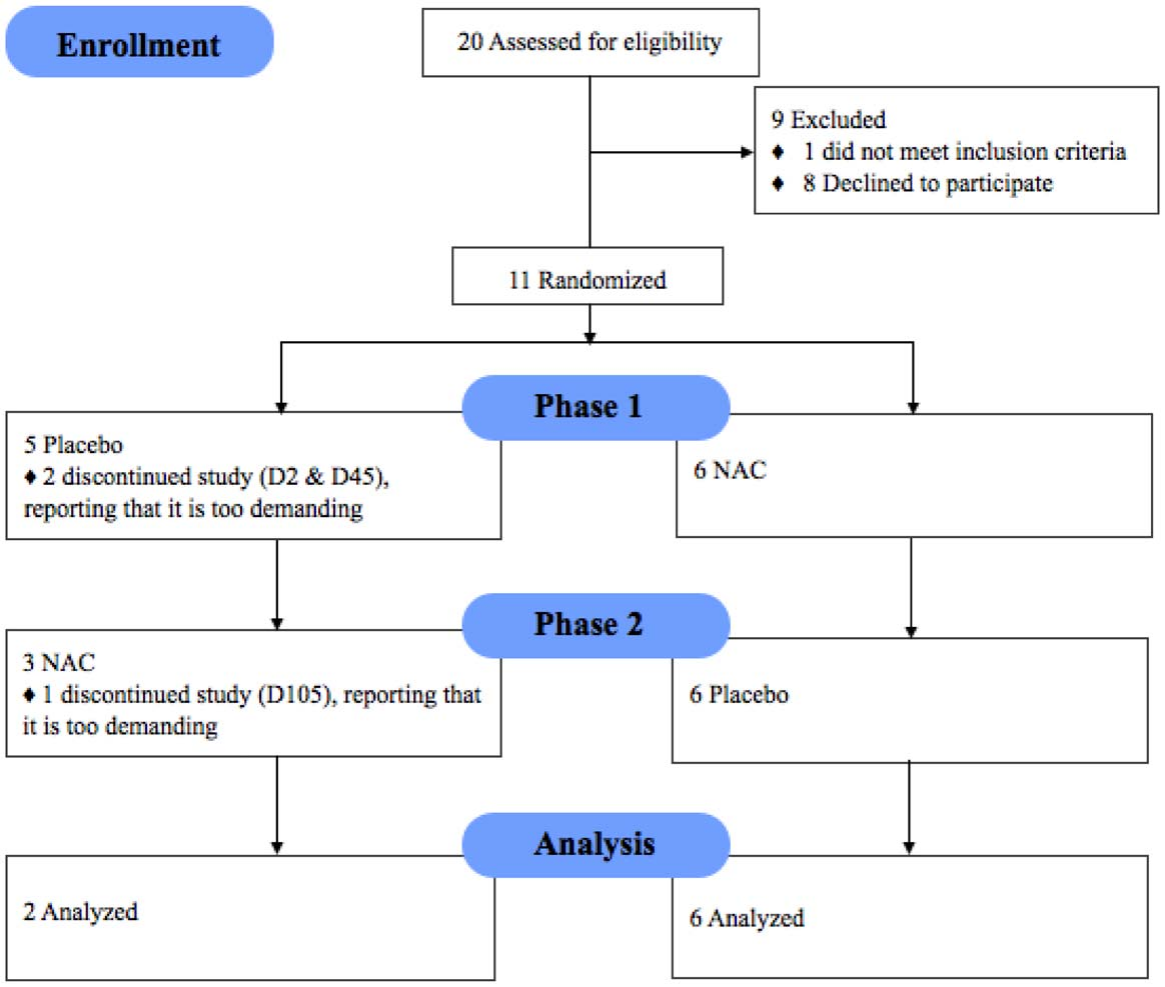
Enrollment, randomization, withdrawals, and completion of the 2 treatment phases (n = 11).

The CONSORT [31] flowchart of the trial is reported in Fig. 1. Eleven patients (nine men; two women; aged 31.9±3.4 years; mean±standard error) meeting DSM-IV criteria for schizophrenia were recruited from the ambulatory Schizophrenia Service of the Department of Psychiatry of the Centre Hospitalier Universitaire Vaudois by an experienced psychiatrist and a psychologist well trained in Diagnostic Interview for Genetic Studies. The mean duration of illness was 9.4±2.5 years. All patients received atypical antipsychotics except one who was drug-naïve. The complete demographic and clinical characteristics of participants were described in [32]. Among the 11 patients, 9 participated in the clinical trial and 8 completed the entire study, including EEG recordings at crossover and at the end of the study. The two patients who withdrew from the study reported that it was too demanding for them. Of the eight patients who completed the entire study, six were in the group that first received NAC and then placebo; the remaining two received placebo first and then NAC. As reported in [29], no side-effects due to NAC have been observed. Both patients and investigators were blinded until the time of analysis, when data pooling necessitated unblinding. Recruited patients gave fully informed written consent, and the Ethics Committee of the Faculty of Biology and Medicine of the University of Lausanne approved all procedures. All 11 patients underwent the Diagnostic Interview for Genetic Studies, developed by the NIMH [33], [34], and remained on their usual antipsychotic medication for the duration of the trial both in terms of type and dose of medication. Recently the effects of NAC on auditory sensory processing as manifested by mismatch negativity, a component of auditory evoked potential related to NMDA receptor function, were studied in this group of patients [32].

#### EEG recording and pre-processing

The resting EEGs were recorded with the 128-channel Geodesic Sensor Net in a semi-dark room with a low level of environmental noise. To control the quality of recordings the EEG tracings were constantly monitored online. The subjects were seated in a comfortable chair and instructed to stay relaxed and motionless with eyes closed for 3–4 min. All the electrode impedances were kept under 30 kΩ [35]. The recordings of vertex-reference EEG were made using a 12-bit analog-to-digital converter, a digitization rate of 1000 samples/s, and a low-pass filter set to 100 Hz. They were further filtered (FIR, band-pass of 1–70 Hz, notch at 50 Hz) and segmented into non-overlapping 1 s epochs using NS 4.2 software (EGI Inc., Eugene, OR, USA). Finally, two separate data sets were obtained for further analysis: one by re-referencing the data against the common average reference (CAR), and one by transforming the data into a two-dimensional surface Laplacian computed via the Current Source Density toolbox [36]. Those two data sets were the input for two separate synchronization analyzes, described further.

Artifacts in all channels were edited offline: first, automatically, based on an absolute voltage threshold (100 µ*V*) and on a transition threshold of 50 µ*V* (sample to sample), and then on the basis of a thorough visual inspection. The sensors that recorded artifactual EEG (>20% of the recording time) were corrected using the bad channel replacement tool (NS 4.2 EGI, USA). Furthermore, we excluded the outer-ring sensors because of their well-known instability. The number of artifact-free epochs entered into the analysis was 171±39 for the NAC condition and 168±62 for Placebo condition.

The data were filtered into four EEG frequency bands: theta (3–7 *Hz*), alpha (7–13 *Hz*), beta (13–30 *Hz*), and gamma (30–48 *Hz*). To this end, we applied an FIR filter with no phase-shift [37].

#### Multivariate phase synchronization

To assess the interactions among the recorded neuronal pools, we computed a Multivariate Phase Synchronization (MPS) index, that is, the modulus of an order parameter of a population of oscillators [38]. MPS is a measure sensitive only to coherence in the phase of oscillations and not to their amplitude. Specifically, MPS measures the amount of phase similarity within a population of oscillators. Consequently, the MPS may be interpreted as the collective rhythm produced by the population under study. For instance, if all oscillators move in a single tight clump, MPS is approximately 1 and the population acts like one giant oscillator. If the oscillations occur incoherently and no macroscopic rhythm is produced, MPS is approximately 0.

Let 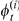 be the sampled phase of oscillator (*i*) at time *t*, *L* the number of available samples, and *N* the number of systems under study. The MPS among the *N* oscillators is given by the formula
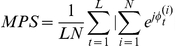
where *j* is the imaginary unit. The first sum provides an instantaneous estimate of the degree of phase similarity. The second sum averages that over time, thus providing an estimate of the synchronization during the observed time period. The MPS was calculated from the pre-processed epoched EEG signals in the four conventional frequency bands (see previous sub-section). The signal phase was estimated by means of the Hilbert transform [39]. To assess the whole-head synchronization topography, we computed the MPS sensor-wise over the cluster of locations defined by the sensor itself and the surrounding sensors belonging to its second-order neighborhood [40]. Such a cluster (on average about 12 *cm* wide) is shown in Fig. S1 and spans typical distances of cortico-cortical connectivity [41]. This approach has been numerically validated and proved to be a robust and easily scalable multivariate approach, highly sensitive even with a reasonably small amount of data [42].

The interpretation of surface EEG synchronization is limited because of its contamination by volume conduction and reference electrode effects [43]. These unwanted effects can be minimized with a high-resolution Laplacian, which isolates the source activity under each sensor [44]. Yet along with volume conduction, a Laplacian removes genuine synchronization of widely distributed source regions, which can be captured by CAR EEG. Here we examined the topography of synchronization based on a combination of Laplacian and CAR EEG, thus encompassing both smaller and intermediate spatial scales of EEG dynamics.

To minimize the effects of signal-to-noise ratio variations on synchronization results, we performed a supplementary analysis of EEG power and removed from the MPS maps those sensors for which such effects could be significant (see Text S1).

### Statistical inference

#### Population Inference

Statistical assessment was performed using the repeated measures one-way ANOVA with two levels (NAC and Placebo). Statistical inference was performed independently for each frequency band of interest. The MPS and Power/Energy estimations that entered into the statistical analysis were computed for each patient by using a summary statistic (the median) over values obtained for all epochs belonging to the same condition. Considering that synchronization and power/energy values vary over a finite interval and thus cannot have a Gaussian distribution, we applied a non-parametric permutation approach. The permutation procedure was refined according to a repeated measure design [45].

A *P*-value for each sensor was obtained by performing 5000 permutations, which were identical for each sensor in order to retain the spatial covariance structure of the data. To get a statistical significance for the whole map, we controlled for multiple hypothesis testing by computing false discovery rates (FDRs) with the BH linear step-up method [46]. As the computation of each MPS value involved its neighbors, the FDR at each sensor was estimated from the *P*-values of the neighboring sensors. All the reported effects correspond to FDR values less than 0.05. To highlight the most pronounced effects, we computed a Cohen index for the size of the effects. Following a conservative statistical approach, we report only sensors with effect size bigger than 1 (i.e. the standard deviation of the effect is bigger than its average value) for CAR EEG, and 0.7 for Laplacian. Finally, we report only clusters composed of at least three neighboring sensors.

#### Individual Inference

To minimize the losses due to a high dropout rate characteristic for a crossover design, we tuned our analysis to a small sample size not only by using extremely rigorous and conservative group statistical analysis, but also by applying an individual level statistics. For each patient, the distribution of the MPS values upon the available epochs was compared between the two conditions, NAC and Placebo. To test the null hypothesis of equal medians, we applied a Wilkoxon rank-sum test. The BH-based FDR values were verified to be at least <0.05, and only sensors significant at population level were kept. Considering that the clinical effects of NAC were already established in a large-sample double-blind, randomized, placebo-controlled trial with 140 patients [29], our approach seems to be sufficient for a neuroimaging description of the probable mechanisms of clinical improvement in at least a subsample of Schizophrenia patients.

#### Correlation analysis

To assess whether the synchronization changes in the patients are related to psychopathological scores, we analyzed the correlation topography between MPS and PANSS as well as between MPS and Liddle's factors [30]. The correlation maps were computed by estimating the Pearson correlation coefficient sensor-wise between the MPS contrast and the corresponding contrast for PANSS and for Liddle's score. To determine whether the correlation values were significantly non-zero, we applied a permutation-based test [47]. The *P*-values obtained after performing 5000 permutations were corrected for multiple comparisons by means of the BH method and considered significant at FDR<0.05.

## Results

**Figure 2:**
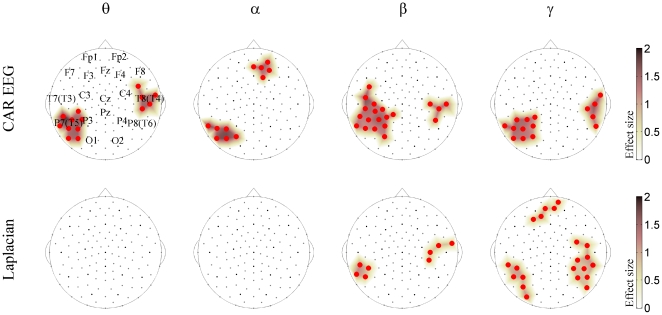
*NAC vs. Placebo* changes in population multivariate EEG synchronization. The whole-head maps for the MPS show the surface topography of the *NAC vs. Placebo* effect. The maps are presented for CAR EEG data (top) and Laplacian data (bottom) for the theta (θ), alpha (α), beta (β), and gamma (γ) frequency bands. They are superimposed on the diagrams of the Geodesic 128-channel Sensor Net. The sensors corresponding to the International 10–20 System are shown with black circles. They are labeled in the upper left diagram. The large circles (irrespective of color) designate significant effect. The red sensors correspond to *NAC>Placebo*. All the effects are shown at FDR<0.05 and the sizes of effects are thresholded at 1 for CAR EEG and 0.7 for Laplacian. The colored surface (obtained by a trilinear interpolation from the three nearest electrodes) represents the effect size (see Materials and Methods for details).

The NAC treatment significantly affected EEG synchronization (Fig. 2). As the head diagrams show, in the CAR EEG, it induced MPS increase between the sensors clustered over three locations – namely, the left parieto-temporal (the neighborhood of T5 and P3 locations), the right temporal (the neighborhood of T4), and the right prefrontal (the neighborhood of Fp2). The left parieto-temporal cluster was significant across all frequencies, the right temporal cluster appeared at the theta and beta-gamma frequencies, while the prefrontal cluster was limited to the alpha band. For Laplacian, we found MPS increases in these three locations only for the higher (beta-gamma) EEG frequencies.

**Figure 3:**
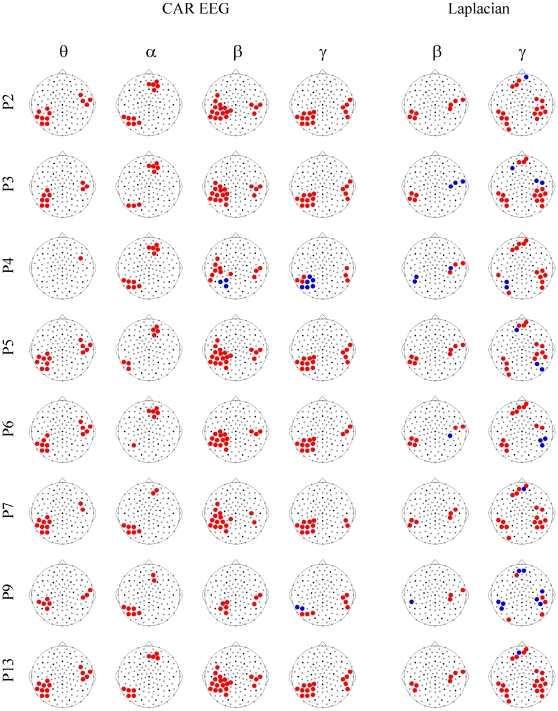
*NAC vs. Placebo* changes in individual multivariate EEG synchronization. The whole-head maps for the MPS show the surface topography of the *NAC vs. Placebo* effect for individual patients. The reported significant changes are restricted to the sensors and frequency bands that demonstrate a significant effect at the population level (Fig. 1), including four frequency bands for CAR EEG (θ, α, β, γ) and two frequency bands for Laplacian (β, γ). Patients are labeled as P2, P3, P4, P5, P6, P7, P9 and P13. Other designations are as in Figs. 2 and S1.

Being limited to a small sample, we secured our group-level findings by considering the individual NAC-induced changes of EEG synchronization (Fig. 3). As can be seen from this figure, the MPS increases in the CAR EEG were robust across all the patients in all the frequency bands, except the theta band in patient P4. In the Laplacian, the results were more variable; yet, for each frequency band, the group-significant effects could be tracked at least in six out of eight patients. It should be noted that a supplementary analysis of EEG energy showed no significant effects of the NAC treatment and that the sensors for which the changes in EEG energy correlated with MPS were outside the clusters shown in Fig. 2. Furthermore, Fig. 3 shows that the imbalance in the number of patients receiving Placebo or NAC first did not affect our findings. Indeed, patients P2 and P5, which received Placebo earlier than NAC, demonstrate responses similar to the average one (Fig. 2). Furthermore, since the majority of the patients received NAC treatment prior to Placebo, any carryover effects would have reduced the difference in synchronization between the two treatment types. The fact that the effect of NAC nonetheless withstands this sequence argues against a strong influence of treatment order.

**Figure 4:**
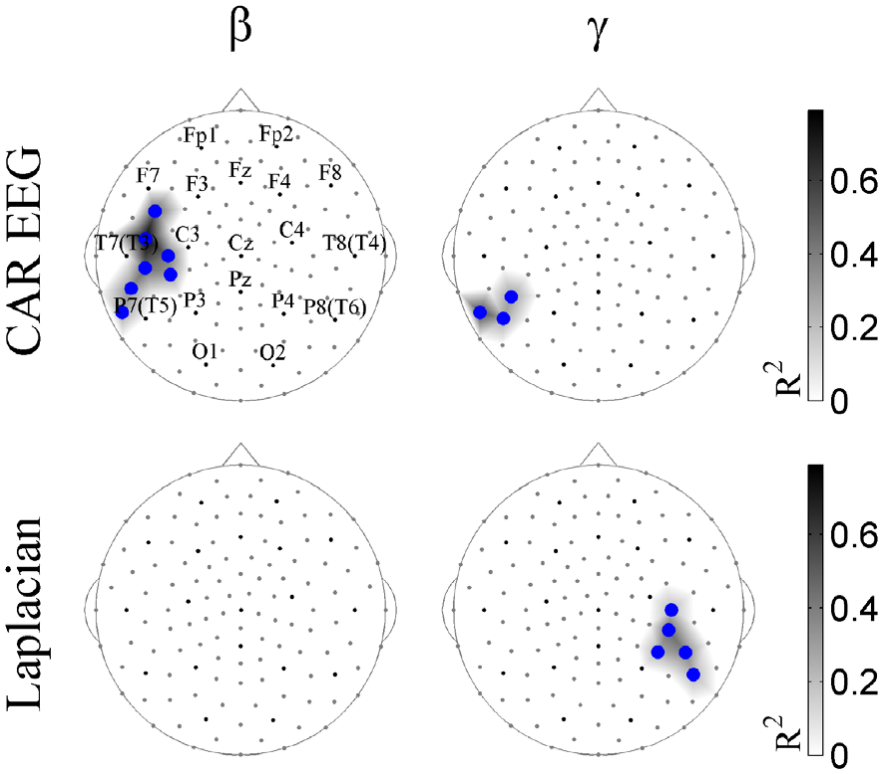
Surface topography of correlations between MPS and Liddle's factor of disorganization for *NAC vs. Placebo* contrast. Significant Pearson correlations at FDR<0.05 are shown in the alpha (α) and beta (β) frequency bands for CAR EEG and Laplacian data. Blue circles designate the sensors with inverse correlations. The size of the correlations is reported as the coefficient of determination defined as the square of the Pearson correlation value (R^2^).

A correlation analysis allowed us to look at the relationship between EEG synchronization and the psychopathological scores of the patients. No correlations were found between MPS and PANSS scores for the contrast *NAC vs. Placebo*. Since various factor analytic studies point to the fact that three syndromes (negative, positive, and disorganization) may underlie schizophrenia symptomatology [48], [49], and that the disorganization syndrome is associated with executive functions and attention [50], [51], we also explored the relationship between EEG synchronization and Liddle's scores [30]. We found significant inverse correlations between Liddle's factor of disorganization and MPS changes in the *NAC vs. Placebo* contrast for the left parieto-temporal cluster at beta and gamma frequencies (Fig. 4). These correlations show that the greater the MPS increase, the greater the clinical improvement. The average value of the coefficient of determination (R^2^) was 0.39 for CAR EEG and 0.31 for Laplacian, which means that synchronization variation can predict or explain 39% and 31% of Liddle's score variation, respectively.

Additionally to the findings of NAC effects on EEG synchronization, we provide a description of placebo effects as seen via the contrast *Placebo>Baseline* (see Text S1 and Figs. S2, S3 and S4).

## Discussion

Our study, through whole-head EEG imaging of multivariate EEG synchronization, revealed for the first time that NAC, a GSH precursor, significantly affects EEG synchronization in schizophrenia patients. In so doing, it increases synchronization for three clusters located over the left parieto-temporal, the right temporal, and the bilateral prefrontal regions. These changes are manifested across theta-gamma frequency bands in the CAR EEG but limited to beta-gamma frequencies in Laplacian, suggesting involvement of deep sources at slow frequencies and both deep and superficial sources at fast frequencies [52], which fits current models of EEG rhythm generation [53]–[57].

The synchronization changes are robust not only at a group, but also at an individual level, which, at least partially, compensates for the limitation imposed by the small size of the sample under analysis. In other words, the significant individual synchronization changes confirm the presence of a subgroup of schizophrenia patients that respond to NAC by changing the landscape of functional connectivity. Correlations between the treatment-related dynamics of Liddle's score for the factor of disorganization and the synchronization changes in the *NAC vs. Placebo* contrast for the left posterior and the bilateral prefrontal clusters further validate these data. These results are also consistent with reports on association between the syndrome of disorganization and defective performance in tasks involving executive functions and attention as well as language and, possibly, body representation [50], [51].

In the context of a specific pattern of synchronization changes, it is important to consider that the participants of this study were among the patients involved in a randomized, double-blind, placebo controlled, add-on clinical trial, in which the GSH precursor NAC significantly improved the negative symptoms and reduced side-effects of antipsychotics [29]. Furthermore, in roughly the same group of patients (7 out of the 8 analyzed here), NAC improved the auditory evoked potential by increasing the NMDA-dependent mismatch negativity [32], which is typically reduced in schizophrenia [58]–[60]. On the whole, the EEG/MEG studies suggest that the temporal and frontal cortices contribute to generation of mismatch negativity (for recent review see [61]), which was the case for this group as well [32]. The remarkable agreement between the topography of mismatch negativity generators, as shown by the source analysis in the latter study, and the clusters of NAC-related changes in multivariate synchronization, revealed here, suggests that functional connectivity plays a role in the central auditory and cognitive dysfunction underlying the schizophrenia symptoms.

Our findings are in agreement with previous research emphasizing abnormal functional connectivity in schizophrenia including resting and task related changes (for review see [62]). The report of Jalili and collaborators [17] is of special interest here, since it provides the whole-head landscape of multivariate synchronization in schizophrenia patients in comparison with normal subjects. Although that study applied a different measure of multivariate synchronization, namely the S-estimator [40], experimental data show similarity in results obtained with different estimators of multivariate EEG synchronization (e.g., [63]), and, thus, allow a qualitative analysis of the NAC-related changes in the context of the resting pattern of synchronization in schizophrenia.

Resting EEG in patients was characterized by bilateral hyper-synchronized clusters over the temporal lobes and by a hypo-synchronized cluster over the midline postcentral region [17]. The individual scores from all the PANSS scales correlated with these abnormalities such that the greater synchronization changes (including increase in synchronization) in all the clusters were linked to the aggravation of pathological symptoms. Furthermore, their topography was similar to the structural maps of cortical damage in schizophrenia [64]. Considering the fact that there is only a partial overlap between the hyper-synchronized temporal clusters in untreated patients [17] and the regions of increased synchronization in the NAC-treated patients (here), we can suggest that NAC is involved in a compensatory response within the networks with relatively preserved connectivity.

As mentioned in the Introduction, our experimental model has shown that abnormal connectivity can result from a redox dysregulation induced by a compromised GSH synthesis. Indeed, in the hippocampus, GSH deficit results in the alterations of NMDA-dependent synaptic plasticity through interaction with the NMDA redox site [13]. Under an oxidative stress, GSH deficit induces a reduction in beta/gamma-oscillations in the ventral hippocampus, associated with social and emotional behavioral anomalies [15]. In the anterior cingulate cortex, transitory GSH deficit during development leads to decreased spine density [8], [65]. Similar findings were reported in schizophrenia patients [66], [67].

These abnormalities in anatomical and functional connectivity are associated with changes in the GABA-ergic fast spiking parvalbumine interneurons. For example, a reduction in beta/gamma-oscillations in ventral hippocampus was associated with a decrease in the number of parvalbumine-immunoreactive GABA interneurons [15]. In the anterior cingulate of *gclm−/−* mice, a decrease in the power of beta and gamma oscillations was accompanied by the delayed maturation of parvalbumine interneurons [68]. This is consistent with the critical role of the GABA-ergic fast spiking parvalbumine interneurons in the mechanism of network synchronization [69]–[73].

A large body of evidence consistently supports the claim that parvalbumine-immunoreactive GABA interneurons are altered in schizophrenia [74]. Moreover, converging facts link this alteration to the hypofunction of NMDA-receptors [75]. Specifically, the hypofunction of NMDA-receptors leads to the over-production of free radicals, which can induce the impairment of parvalbumine interneurons [76], [77]. Considering the association between NMDA-receptors' hypofunction and GSH deficit, these results suggest that the GSH precursor NAC can improve synchronization dynamics in schizophrenia through interaction with parvalbumine GABA-ergic connectivity [2].

Indeed, here we observed such effects of NAC on synchronization after 2 months of treatment, at a time when no significant amelioration of schizophrenia symptoms was yet detected at a group level. However, (i) in this group, the Liddle disorganization factor scores correlated with the individual parieto-temporal synchronization responses to NAC, which survived rigorous statistical testing, and (ii) the clinical picture improved in a larger sample within the frame of a six-month clinical study [29]. In the case that the results observed in the present proof of concept study can be reproduced within a larger clinical sample, we conclude that synchronization dynamics can be more sensitive to the treatment effects than conventional symptomatology scales and that it has potential as an early biomarker for treatment efficacy.

## Supporting Information

Figure S1 **Example of spatial localization of MPS estimator.** The sensor locations in red exemplify the second neighborhood for sensor in green (sensor 76) that is the territory considered in the calculation of a single value of MPS.
(TIF)

Figure S2 ***Placebo vs. Baseline*** **changes in population multivariate EEG synchronization.** The whole-head maps for the MPS show the surface topography of the *Placebo vs. Baseline* effect in the whole group of schizophrenia patients. The significant effects were obtained for CAR EEG (top) and Laplacian (bottom) for the theta (θ), alpha (α) and beta (β) frequency bands. They are superimposed on the diagrams of the Geodesic 128-channel Sensor Net. The sensors corresponding to the International 10–20 System are shown with black circles. They are labeled in the upper left diagram. The large circles (irrespective of color) designate significant effect. The red sensors correspond to *Placebo>Baseline*, while the blue sensors correspond to *Placebo<Baseline*. All the effects are shown at FDR<0.05. The colored surface (obtained by a trilinear interpolation from the three nearest electrodes) represents the effect size (see Materials and Methods for details). The significant effects are thresholded at an effect size of value equal to 1.
(TIF)

Figure S3 ***Placebo vs. Baseline*** **changes in individual multivariate EEG synchronization.** The whole-head maps for the MPS show the surface topography of the *Placebo vs. Baseline* effect for individual patients. The reported significant changes are restricted to the sensors and frequency bands that demonstrate a significant effect at the population level (Fig. S1), including three frequency bands for CAR EEG (θ, α, β) and two frequency bands for Laplacian (θ, β). Patients are labeled as P2, P4, P5, P6, P7, P9 and P13. P3 is missing here since baseline recording was of insufficient quality. Other designations are as in Figs. 2 and S1.
(TIF)

Figure S4 **Surface topography of correlations between MPS and Liddle's factor of disorganization for** ***Placebo vs. Baseline*** **contrast.** Significant Pearson correlations at FDR<0.05 obtained in the theta (θ) and beta (β) frequency bands for CAR EEG are shown with large red (for positive correlations) circles. The size of correlations is reported with the coefficient of determination (R^2^).
(TIF)

Text S1 **Analysis of EEG energy and effects of placebo on EEG synchronization.**
(DOC)

Protocol S1 **Trial protocol 106-03 CE_18-8-03.**
(PDF)

Checklist S1 **CONSORT checklist.**
(DOC)

## References

[1] Do KQ, Bovet P, Cabungcal JH, Conus P, Gysin R, Lajtha A (2009). Redox dysregulation in schizophrenia: genetic susceptibility and pathophysiological mechanisms.. Handbook of Neurochemistry and Molecular Neurobiology, Vol 27.

[2] Do KQ, Cabungcal JH, Frank A, Steullet P, Cuenod M (2009). Redox dysregulation, neurodevelopment, and schizophrenia.. Curr Opin Neurobiol.

[3] Do KQ, Trabesinger AH, Kirsten-Kruger M, Lauer CJ, Dydak U (2000). Schizophrenia: glutathione deficit in cerebrospinal fluid and prefrontal cortex in vivo.. Eur J Neurosci.

[4] Matsuzawa D, Hashimoto K (2011). Magnetic resopnance spectroscopy study of the antioxidant defense system in Schizophrenia.. Antioxid Redox Signal.

[5] Yao JK, Leonard S, Reddy R (2006). Altered glutathione redox state in schizophrenia.. Dis Markers.

[6] Gysin R, Kraftsik R, Sandell J, Bovet P, Chappuis C (2007). Impaired glutathione synthesis in schizophrenia: Convergent genetic and functional evidence.. Proc Natl Acad Sci U S A.

[7] Tosic M, Gysin R, Ott J, Barral S, Bovet P (2006). Schizophrenia and oxidative stress: Glutamate cysteine ligase modifier as a susceptibility gene.. Am J Hum Genet.

[8] Cabungcal JH, Nicolas D, Kraftsik R, Cuenod M, Do KQ (2006). Glutathione deficit during development induces anomalies in the rat anterior cingulate GABAergic neurons: Relevance to schizophrenia.. Neurobiol Dis.

[9] Cabungcal JH, Preissmann D, Delseth C, Cuenod M, Do KQ (2007). Transitory glutathione deficit during brain development induces cognitive impairment in juvenile and adult rats: relevance to schizophrenia.. Neurobiol Dis.

[10] Castagne V, Cuenod M, Do KQ (2004). An animal model with relevance to schizophrenia: sex-dependent cognitive deficits in osteogenic disorder-Shionogi rats induced by glutathione synthesis and dopamine uptake inhibition during development.. Neuroscience.

[11] Castagne V, Rougemont M, Cuenod M, Do KQ (2004). Low brain glutathione and ascorbic acid associated with dopamine uptake inhibition during rat's development induce long-term cognitive deficit: relevance to schizophrenia.. Neurobiol Dis.

[12] Rougemont M, Do KQ, Castagne V (2003). A new model of glutathione deficit during development: effect of glutathione deficit on lipid peroxidation in the rat brain.. J Neurosci Res.

[13] Steullet P, Neijt HC, Cuenod M, Do KQ (2006). Synaptic plasticity impairment and hypofunction of NMDA receptors induced by glutathione deficit: relevance to schizophrenia.. Neuroscience.

[14] Steullet P, Lavoie S, Guidi R, Kraftsik R, Cuenod M (2008). Intracellular glutathione deficit alters dopamine modulation of L-type calcium channels via D2 and ryanodyne receptors.. Free Radic Biol Med.

[15] Steullet P, Cabungcal JH, Kulak A, Kraftsik R, Chen Y (2010). Redox Dysregulation Affects the Ventral But Not Dorsal Hippocampus: Impairment of Parvalbumin Neurons, Gamma Oscillations, and Related Behaviors.. J Neurosci.

[16] Boutros NN, Arfken C, Galderisi S, Warrick J, Pratt G (2008). The status of spectral EEG abnormality as a diagnostic test for schizophrenia.. Schizophr Res.

[17] Jalili M, Lavoie S, Deppen P, Meuli R, Do KQ (2007). Dysconnection topography in schizophrenia revealed with state-space analysis of EEG.. PLoS One.

[18] Koenig T, Lehmann D, Saito N, Kuginuki T, Kinoshita T (2001). Decreased functional connectivity of EEG theta-frequency activity in first-episode, neuroleptic-naïve patients with schizophrenia: preliminary results.. Schizophr Bull.

[19] Rutter L, Carver FW, Holroyd T, Nadar SR, Mitchell-Francis J (2009). Magnetoencephalographic gamma power reduction in patients with schizophrenia during resting condition.. Hum Brain Mapp.

[20] Sponheim SR, Clementz BA, Iacono WG, Beiser M (2000). Clinical and biological concomitants of resting EEG power abnormalitites in schizophrenia.. Biol Psychiatry.

[21] Uhlhaas PJ, Linden DE, Singer W, Haenschel C, Lindner M (2006). Dysfunctional long-range coordination of neural activity during Gestalt perception in schizophrenia.. J Neurosci.

[22] Basar-Eroglu C, Schmiedt-Fehr C, Mathes B, Zimmermann J, Brand A (2008). Are oscillatory brain responses generally reduced in schizophrenia during long sustained attentional processing?. Int J Psychophysiol.

[23] Flynn G, Alexander D, Harris A, Whitford T, Wong W (2008). Increased absolute magnitude of gamma synchrony in first-episode psychosis.. Schizophr Res.

[24] Haenschel C, Linden D (2010). Exploring phenotypes with EEG: working memory dysfunction in schizophrenia.. Behav Brain Res.

[25] De Vico Fallani F, Maglione A, Babiloni F, Mattia D, Astolfi L (2010). Cortical network analysis in patients affected by schizophrenia.. Brain Topogr.

[26] Bachman P, Kim J, Yee CM, Therman S, Manninen M (2008). Abnormally high EEG alpha synchrony during working memory maintenance in twins discordant for schizophrenia.. Schizophr Res.

[27] Bob P, Susta M, Glaslova K, Boutros NN (2010). Dissociative symptoms and interregional EEG cross-correlations in paranoid schizophrenia.. Psychiatric Res.

[28] Uhlhaas PJ, Singer W (2010). Abnormal neural oscillations and synchrony in schizophrenia.. Nat Rev Neurosci.

[29] Berk M, Copolov D, Dean O, Lu K, Jeavons S (2008). N-Acetyl Cysteine as a Glutathione precursor for schizophrenia – a double-blind, randomized, placebo-controlled trial.. Biol Psychiatry.

[30] Liddle PF (1987). Schizophrenic syndromes, cognitive performance and neurological dysfunction.. Psychol Med.

[31] Schulz KF, Altman DG, Moher D, for the CONSORT Group (2010). CONSORT 2010 Statement: updated guidelines for reporting parallel group randomised trials.. BMJ.

[32] Lavoie S, Murray MM, Deppen P, Knyazeva MG, Berk M (2008). Glutathione precursor, N-acetyl-cysteine, improves mismatch negativity in schizophrenia patients.. Neuropsychopharm.

[33] Nurnberger JI, Blehar MC, Kaufmann CA, York-Cooler C, Simpson SG (1994). Diagnostic interview for genetic studies. Rationale, unique features, and training. NIMH for genetics initiative.. Arch Gen Psychiatry.

[34] Preisig M, Fenton BT, Matthey ML, Berney A, Ferrero F (1999). Diagnostic interview for genetic studies (DIGS): inter-rater and test-retest reliability of the French version.. Eur Arch Psychiatry Clin Neurosci.

[35] Ferree TC, Luu P, Russell GS, Tucker DM (2001). Scalp electrode impedance, infection risk, and EEG data quality.. Clin Neurophysiol.

[36] Kayser J, Tenke CE (2006). Principal components analysis of Laplacian waveforms as a generic method for identifying ERP generator patterns: I. Evaluation with auditory oddball tasks.. Clin Neurophysiol.

[37] Selesnick IW, Lang MIW, Burrus CS (1996). Constrained Least Square Design of FIR Filters without Specified Transition Bands.. IEEE Trans Signal Process.

[38] Strogatz SH (2000). From Kuramoto to Crawford: exploring the onset of synchronization in populations of coupled oscillators.. Physica D.

[39] Pikovsky A, Rosenblum MG, Kurths J (2001). Synchronization: a universal concept in nonlinear sciences.

[40] Carmeli C, Knyazeva MG, Innocenti GM, De Feo O (2005). Assessment of EEG synchronization based on state-space analysis.. Neuroimage.

[41] Schuz A, Braitenberg V, Schuz A, Miller R (2002). The human cortical white matter: Quantitative aspects of cortico-cortical long-range connectivity.. Cortical areas: Unity and diversity.

[42] Carmeli C (2006). Assessing cooperative behavior in dynamical networks with applications to brain data.. http://library.epfl.ch/theses/?nr=3651.

[43] Nunez PL, Srinivasan R, Westdorp AF, Wijesinghe RS, Tucker D (1997). EEG coherency I: Statistics, reference electrode, volume conduction, Laplacians, cortical imaging, and interpretation at multiple scales.. Electroencephalogr Clin Neurophysiol.

[44] Srinivasan R, Winter WR, Ding J, Nunez PL (2007). EEG and MEG coherence: measures of functional connectivity at distinct spatial scales of neocortical dynamics.. J Neurosci Methods.

[45] Knyazeva MG, Carmeli C, Fornari E, Meuli R, Small M (2010). Binding under Conflict Conditions: State-Space Analysis of Multivariate EEG Synchronization.. J Cogn Neurosci.

[46] Benjamini Y, Hochberg Y (1995). Controlling the false discovery rate – a practical and powerful approach to multiple testing.. J R Statist Soc B.

[47] Higgins JJ (2004). Introduction to modern nonparametric statistics.

[48] Andreasen NC, Arndt S, Alliger R, Miller D, Flaum M (1995). Symptoms of schizophrenia. Methods, meanings, and mechanisms.. Arch Gen Psychiatry.

[49] Peralta V, de LJ, Cuesta MJ (1992). Are there more than two syndromes in schizophrenia? A critique of the positive-negative dichotomy.. Br J Psychiatry.

[50] Basso MR, Nasrallah HA, Olson SC, Bornstein RA (1998). Neuropsychological correlates of negative, disorganized and psychotic symptoms in schizophrenia.. Schizophr Res.

[51] Ngan ET, Liddle PF (2000). Reaction time, symptom profiles and course of illness in schizophrenia.. Schizophr Res.

[52] Nunez P, Srinivasan R (2006). Electric fields of the brain: the neurophysics of EEG.

[53] Niedermeyer E (1997). Alpha rhythms as physiological and abnormal phenomena.. Int J Psychophysiol.

[54] Hughes SW, Crunelli V (2005). Thalamic mechanisms of EEG alpha rhythms and their pathological implications.. Neuroscientist.

[55] Bartos M, Vida I, Jonas P (2007). Synaptic mechanisms of synchronized gamma oscillations in inhibitory interneuron networks.. Nat Rev Neurosci.

[56] Colgin LL, Moser EI (2010). Gamma oscillations in the hippocampus.. Physiology (Bethesda).

[57] Pignatelli M, Beyeler A, Leinekugel X (2011). Neural circuits underlying the generation of theta oscillations.. J Physiol Paris.

[58] Javitt DC, Doneshka P, Zylberman I, Ritter W, Vaughan HG (1993). Impairment of early cortical processing in schizophrenia: an event- related potential confirmation study.. Biol Psychiatry.

[59] Shelley AM, Ward PB, Catts SV, Michie PT, Andrews S (1991). Mismatch negativity: an index of a preattentive processing deficit in schizophrenia.. Biol Psychiatry.

[60] Umbricht D, Schmid L, Koller R, Vollenweider FX, Hell D (2000). Ketamine-induced deficits in auditory and visual context-dependent processing in healthy volunteers: implications for models of cognitive deficits in schizophrenia.. Arch Gen Psychiatry.

[61] Näätänen R, Kähkönen S (2009). Central auditory dysfunction in schizophrenia as revealed by the mismatch negativity (MMN) and its magnetic equivalent MMNm: a review.. Int J Neuropsychopharmacol.

[62] Uhlhaas PJ, Singer W (2006). Neural synchrony in brain disorders: relevance for cognitive dysfunctions and pathophysiology.. Neuron.

[63] Knyazeva MG, Jalili M, Brioschi A, Bourquin I, Fornari E (2010). Topography of EEG multivariate phase synchronization in early Alzheimer's disease.. Neurobiol Aging.

[64] Thompson PM, Vidal C, Giedd JN, Gochman P, Blumenthal J (2001). Mapping adolescent brain change reveals dynamic wave of accelerated gray matter loss in very early-onset schizophrenia.. Proc Natl Acad Sci USA.

[65] Grima G, Benz B, Parpura V, Cuénod M, Do KQ (2003). Dopamine-induced oxidative stress in neurons with glutathione deficit: implication for schizophrenia.. Schizophr Res.

[66] Glantz LA, Lewis DA (2000). Decreased dendritic spine density on prefrontal cortical pyramidal neurons in schizophrenia.. Arch Gen Psychiatry.

[67] Harrison PJ (1999). The neuropathology of schizophrenia. A critical review of the data and their interpretation.. Brain.

[68] Steullet P, Cabungcal JH, Cuénod M, Do KQ (2010).

[69] Gonzalez-Burgos G, Lewis DA (2008). GABA neurons and the mechanisms of network oscillations: implications for understanding cortical dysfunction in schizophrenia.. Schizophr Bull.

[70] Gonzalez-Burgos G, Hashimoto T, Lewis DA (2010). Alterations of cortical GABA neurons and network oscillations in schizophrenia.. Curr Psychiatry Rep.

[71] Sohal VS, Zhang F, Yizhar O, Deisseroth K (2009). Parvalbumin neurons and gamma rhythms enhance cortical circuit performance.. Nature.

[72] Traub RD, Bibbig A, Lebeau FE, Buhl EH, Whittington MA (2004). Cellular mechanisms of neuronal population oscillations in the hippocampus in vitro.. Annu Rev Neurosci.

[73] Whittington MA, Cunningham MO, LeBeau FE, Racca C, Traub RD (2011). Multiple origins of the cortical gamma rhythm.. Dev Neurobiol.

[74] Lewis DA, Hashimoto T, Volk DW (2005). Cortical inhibitory neurons and schizophrenia.. Nat Rev Neurosci.

[75] Kantrowitz JT, Javitt DC (2010). N-methyl-d-aspartate (NMDA) receptor dysfunction or dysregulation: the final common pathway on the road to schizophrenia?. Brain Res Bull.

[76] Behrens MM, Ali SS, Dao DN, Lucero J, Shekhtman G (2007). Ketamine-induced loss of phenotype of fast-spiking interneurons is mediated by NADPH-oxidase.. Science.

[77] Behrens MM, Sejnowski TJ (2009). Does schizophrenia arise from oxidative dysregulation of parvalbumin-interneurons in the developing cortex?. Neuropharmacology.

