# Supplementary figures and images for: Glutathione Precursor N-Acetyl-Cysteine Modulates EEG Synchronization in Schizophrenia Patients: A Double-Blind, Randomized, Placebo-Controlled Trial
Source: PLoS One. 2012 Feb 22;7(2):e29341. doi: 10.1371/journal.pone.0029341 (PMC3285150; doi:10.1371/journal.pone.0029341)

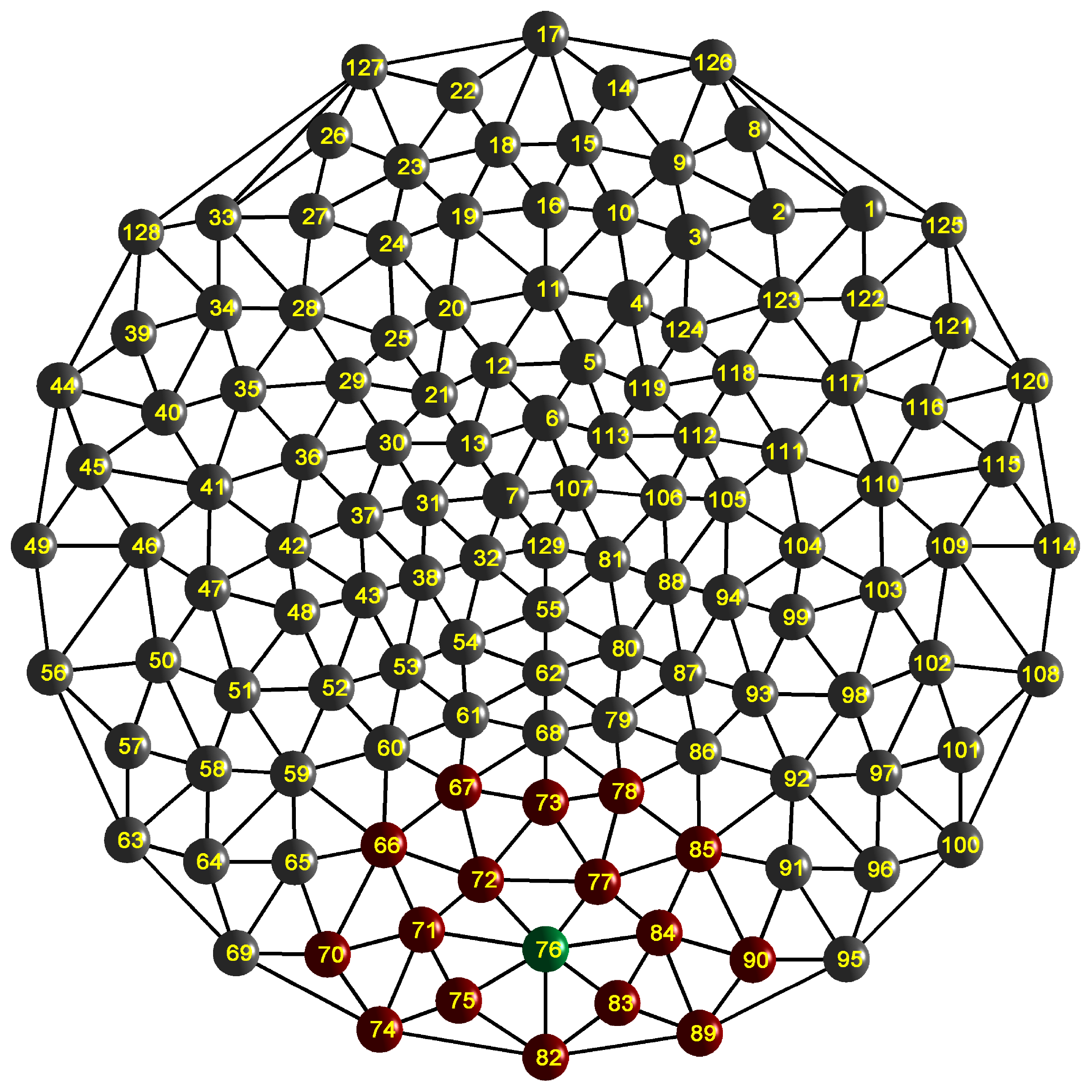

Supplement: Figure S1 — Example of spatial localization of MPS estimator. The sensor locations in red exemplify the second neighborhood for sensor in green (sensor 76) that is the territory considered in the calculation of a single value of MPS. (TIF) [file pone.0029341.s001.tif]

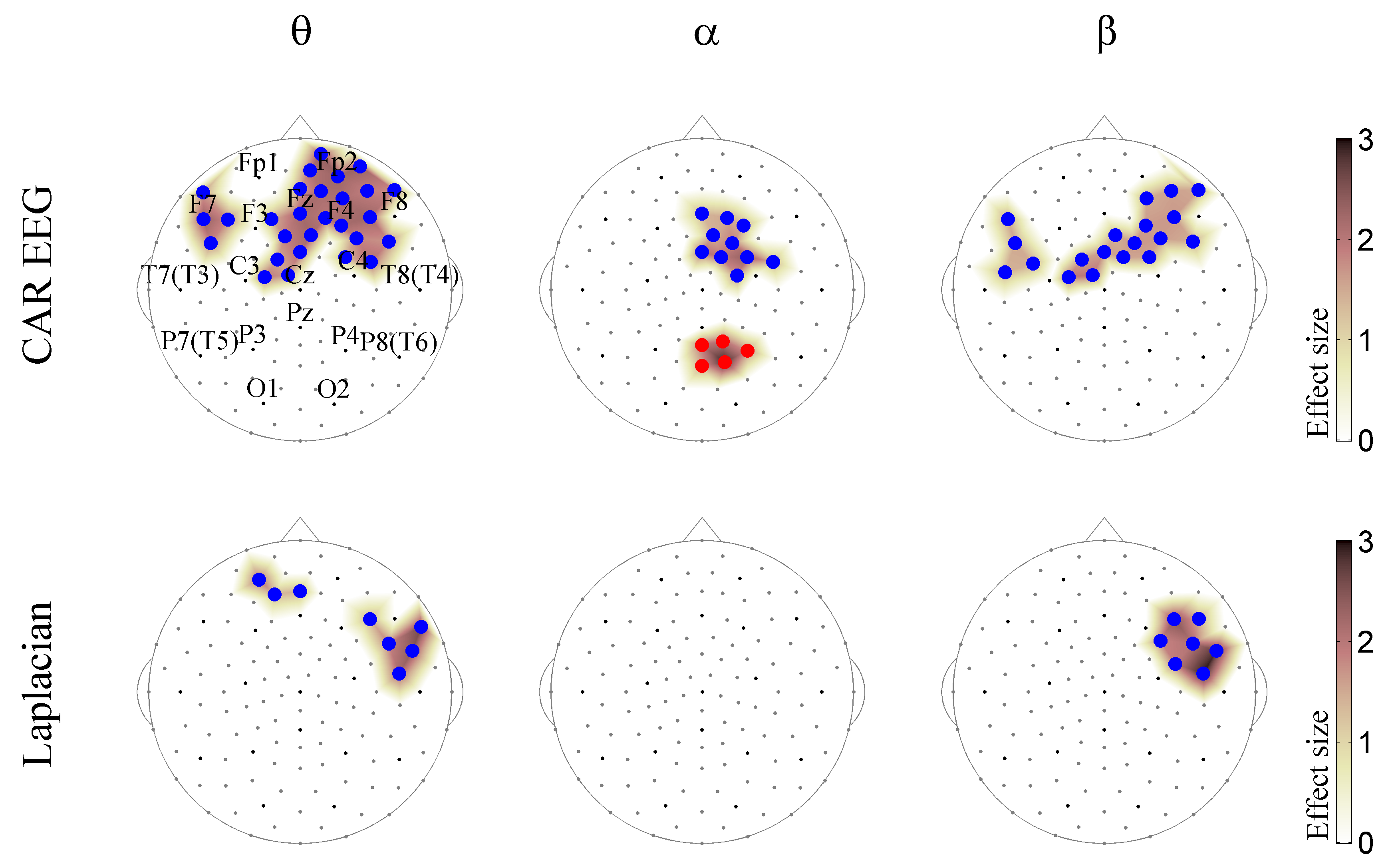

Supplement: Figure S2 — Placebo vs. Baseline changes in population multivariate EEG synchronization. The whole-head maps for the MPS show the surface topography of the Placebo vs. Baseline effect in the whole group of schizophrenia patients. The significant effects were obtained for CAR EEG (top) and Laplacian (bottom) for the theta (θ), alpha (α) and beta (β) frequency bands. They are superimposed on the diagrams of the Geodesic 128-channel Sensor Net. The sensors corresponding to the International 10–20 System are shown with black circles. They are labeled in the upper left diagram. The large circles (irrespective of color) designate significant effect. The red sensors correspond to Placebo>Baseline, while the blue sensors correspond to Placebo<Baseline. All the effects are shown at FDR<0.05. The colored surface (obtained by a trilinear interpolation from the three nearest electrodes) represents the effect size (see Materials and Methods for details). The significant effects are thresholded at an effect size of value equal to 1. (TIF) [file pone.0029341.s002.tif]

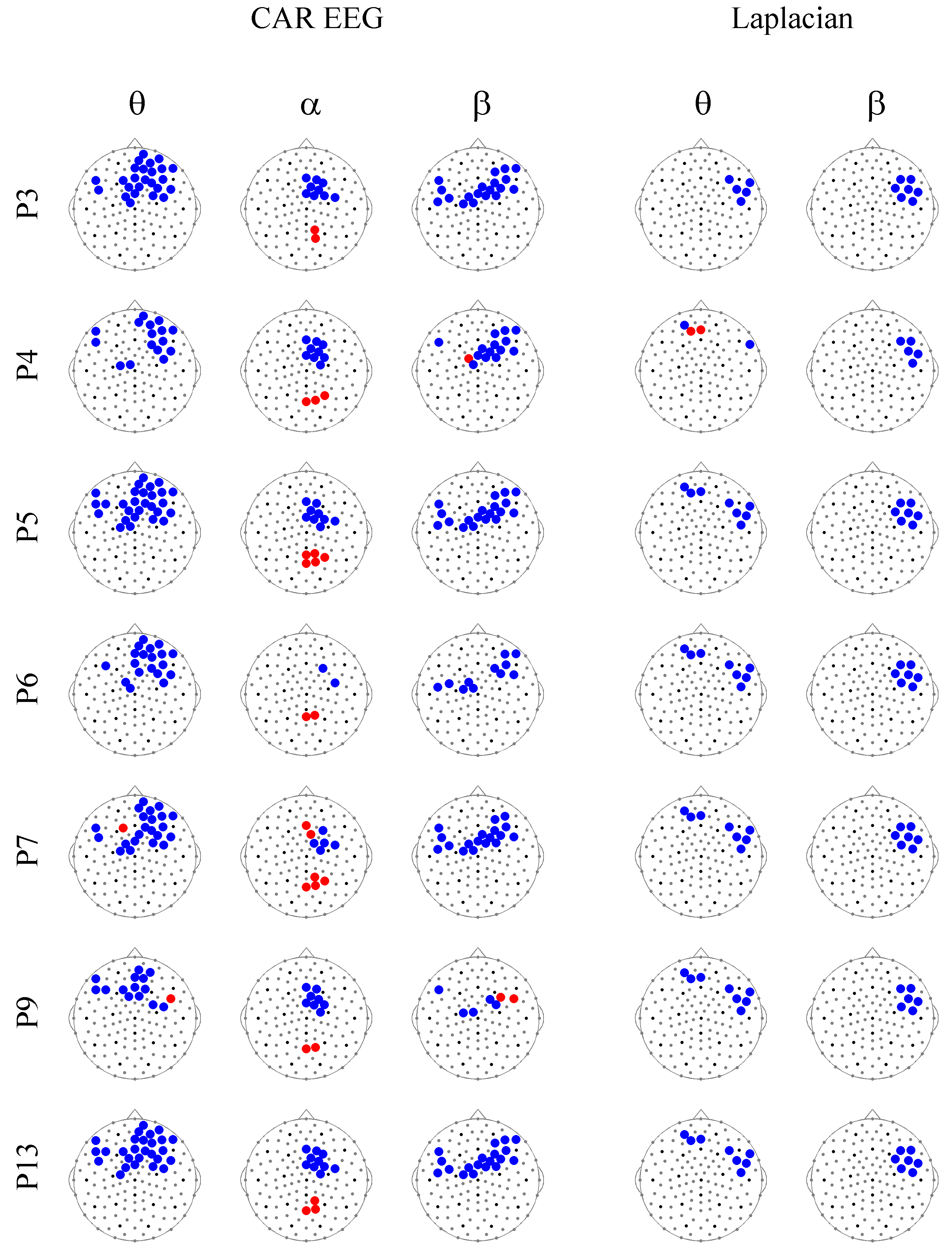

Supplement: Figure S3 — Placebo vs. Baseline changes in individual multivariate EEG synchronization. The whole-head maps for the MPS show the surface topography of the Placebo vs. Baseline effect for individual patients. The reported significant changes are restricted to the sensors and frequency bands that demonstrate a significant effect at the population level (Fig. S1), including three frequency bands for CAR EEG (θ, α, β) and two frequency bands for Laplacian (θ, β). Patients are labeled as P2, P4, P5, P6, P7, P9 and P13. P3 is missing here since baseline recording was of insufficient quality. Other designations are as in Figs. 2 and S1. (TIF) [file pone.0029341.s003.tif]

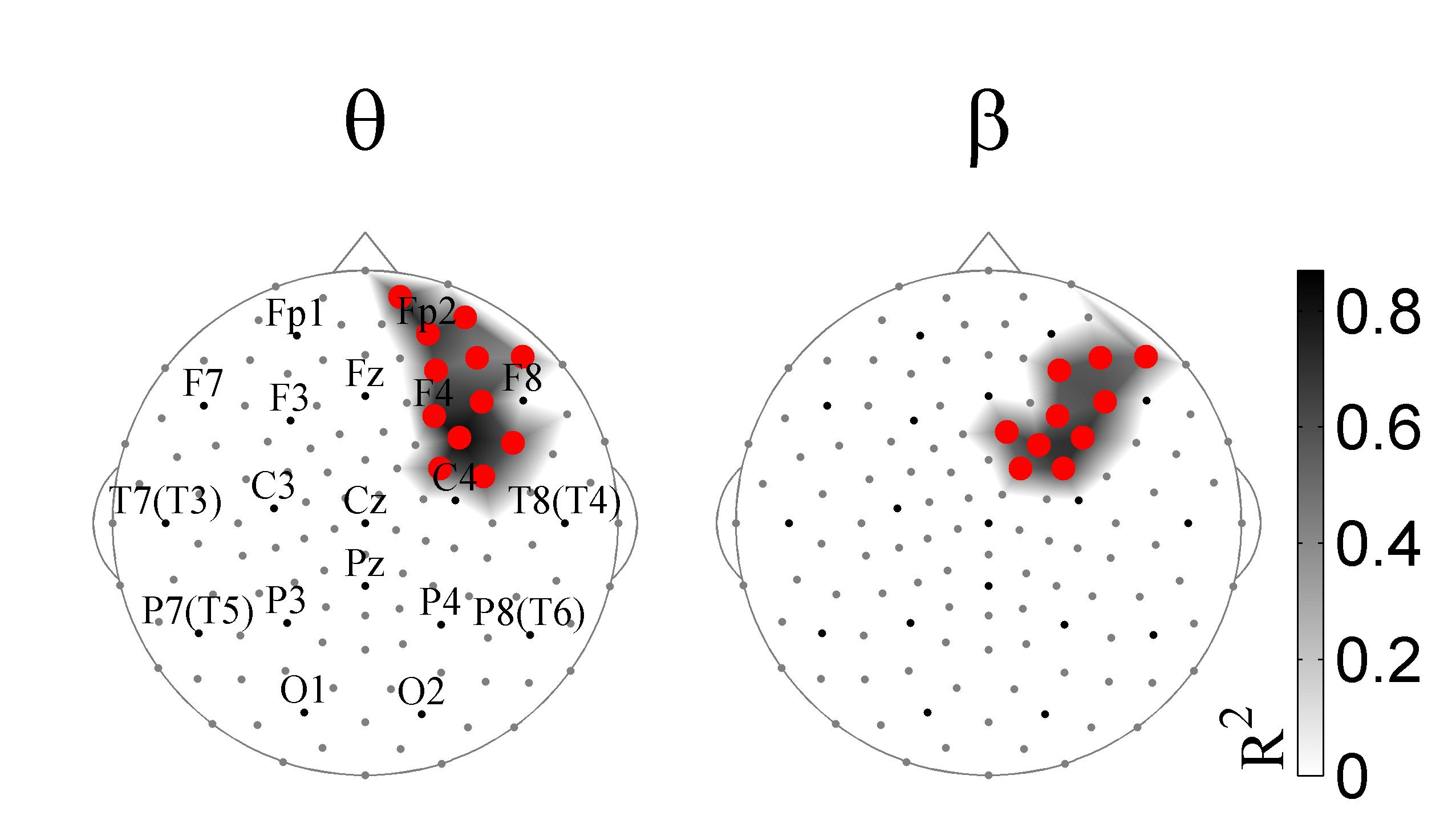

Supplement: Figure S4 — Surface topography of correlations between MPS and Liddle's factor of disorganization for Placebo vs. Baseline contrast. Significant Pearson correlations at FDR<0.05 obtained in the theta (θ) and beta (β) frequency bands for CAR EEG are shown with large red (for positive correlations) circles. The size of correlations is reported with the coefficient of determination (R2). (TIF) [file pone.0029341.s004.tif]
