# Supplementary material for: Glutathione Precursor N-Acetyl-Cysteine Modulates EEG Synchronization in Schizophrenia Patients: A Double-Blind, Randomized, Placebo-Controlled Trial
Source: PLoS One. 2012 Feb 22;7(2):e29341. doi: 10.1371/journal.pone.0029341 (PMC3285150; doi:10.1371/journal.pone.0029341)
Supplement: Text S1 — Analysis of EEG energy and effects of placebo on EEG synchronization. (DOC) [file pone.0029341.s005.doc]

***Analysis of EEG energy.*** To exclude a possibility that the reported changes in MPS are the side effects of modulations in signal-to-noise ratio rather than in synchronization as such, we performed a supplementary analysis of EEG energy. We computed the power spectra with the multi-taper method [1]. This method involves the multiplication of data segments with multiple tapers before Fourier transformation and effectively concentrates spectral estimates across a specified frequency band. We used a set of tapers to achieve a spectral concentration over +/- 3 *Hz*. Eventually, average power in a frequency band of interest was computed via an integral of power spectral density for each subject, sensor, and condition. Based on these values, whole-head energy maps were constructed.

To compare the topography of quantitative changes in synchronization with the topography of quantitative changes in EEG energy for each sensor, we computed the Pearson correlation coefficients between the changes in these two indices. The statistical procedure was analogous to the one used for the correlation analysis with behavioral indices. Overall, 8 ± 5 (mean ± SD) sensors showed significant correlations at *FDR* < 0.05. To minimize the effects of power on the synchronization results, we removed such sensors from MPS maps.

***Effects of placebo on EEG synchronization.*** Nearly nothing is known about the neurophysiological correlates of brain mechanisms that mediate placebo effects in schizophrenia [2]. In our patients’ group, although there was no significant placebo effect at a group level, the placebo treatment significantly changed EEG synchronization compared to baseline condition (Fig. S2) and correlated with clinical outcome (Fig. S4). In CAR EEG, it induced an MPS decrease between the sensors clustered over the prefrontal and precentral areas of both hemispheres at the theta and beta frequencies. In Laplacian, such clusters were predominantly located over the right fronto-temporal region. At alpha frequencies we have found a different pattern that consists of two clusters over the midline/right hemisphere. The prefrontal hypo-synchronized cluster was located roughly between F4 and C4, whereas parietal hyper-synchronized cluster was between P2 and P4. The topography of these clusters together with their absence in the surface Laplacian suggest involvement of deep medial cortical structures, probably cingulate.

The individual changes in synchronization (Fig. S3) strongly confirm the placebo-induced hypo-synchronization over the right prefrontal cortex at theta and beta frequencies across CAR EEG and Laplacian, as well as the changes obtained for alpha activity. Moreover, a correlation analysis for *Placebo vs. Baseline contrast* showed that the weakening of the disorganization syndrome measured with Liddle’s factor of disorganization was associated with MPS decrease over the right frontal cortex (Fig. S4). In particular, according to the mean value of the coefficient of determination (R^2^), synchronization variation explains 61% of Liddle’s score variation.

Our findings suggest an involvement of top-down prefrontal influences in the placebo effects in schizophrenia. They are consistent with the significant role of prefrontal cortex that was repeatedly shown in placebo analgesia [3] and in placebo effects in various mental and behavioral disorders [4-6].

**References**

1. Mitra PP, Pesaran B (1999) Analysis of dynamic brain imaging data. Biophys J 76: 691-708.

2. Benedetti F (2009) Placebo effects. Understanding the mechanisms in health and disease. Oxford: Oxford University Press. 291 p.

3. Petrovic P, Kalso E, Petersson KM, Andersson J, Fransson P, et al. (2010) A prefrontal non-opioid mechanism in placebo analgesia. Pain 150:59-65.

4. Diederich NJ, Goetz CG (2008) The placebo treatments in neurosciences: New insights from clinical and neuroimaging studies. Neurology 71: 677–684.

5. Oken BS (2008) Placebo effects: clinical aspects and neurobiology. Brain 131: 2812-2823.

6. Zubieta JK, Stohler CS (2009) Neurobiological mechanisms of placebo responses. Ann NY Acad Sci 198-210.
