# Supplementary material for: Glutathione Precursor N-Acetyl-Cysteine Modulates EEG Synchronization in Schizophrenia Patients: A Double-Blind, Randomized, Placebo-Controlled Trial
Source: PLoS One. 2012 Feb 22;7(2):e29341. doi: 10.1371/journal.pone.0029341 (PMC3285150; doi:10.1371/journal.pone.0029341)
Supplement: Protocol S1 — Trial protocol 106-03 CE_18-8-03. (PDF) [file pone.0029341.s006.pdf]

DEPARTEMENT UNIVERSITAIRE
DE PSYCHIATRIE ADULTE

HOSPICES CANTONAUX /
ETAT DE VAUD

CHEF DE DEPARTEMENT
PROF. PATRICE GUEX

Protocole N0 106/03 CE

EFFETS DE LA N-ACETYLCYSTEINE (NAC) EN PRISE ORALE SUR LE TAUX DE GLUTHATION ET LES SYMPTOMES DES TROUBLES DU SPECTRE DE LA SCHIZOPHRENIE: ETUDE EN DOUBLE AVEUGLE ET CROSS-OVER.

Correspondance:
PD Dr Kim Q. Do-Cuénod

Centre de Neurosciences Psychiatriques
Site de Cery
CH- 1008 PRILLY-LAUSANNE
Tél: +41-21-643 65 65
Fax: +41-21-643 65 62
E-mail:Kim.Do@inst.hospvd.ch

SOMMAIRE

1) Résumé	3
2) Durée de l'étude	3
3) Financement	3
4) Investigateurs	4
5) Etat actuel de la recherche	5
6) Médicament de l'étude	7
7) Objectifs de l'étude	9
8) Méthodologie : plan de l'étude, sujets et investigations	10
(a) Durée de l'étude	11
(b) Sélection des sujets	12
(c) Schéma général	13
(d) Méthodes d'investigation	14
9) Questions éthiques	19
10) Bibliographie	21

1. RÉSUMÉ

Les résultats actuels de l'étude « Schizophrénie, troubles apparentés et glutathion » renforcent l'hypothèse proposée qu'un déficit intracérébral en glutathion est un facteur de vulnérabilité pour la schizophrénie, au moins pour un sous-groupe de patients. Tout en poursuivant cette étude, il est justifié maintenant de tenter de rétablir un niveau plus élevé de glutathion chez des patients afin de voir si cette élévation s'accompagne d'une amélioration des symptômes, en particulier des symptômes négatifs et des troubles cognitifs, particulièrement résistants à la thérapie actuelle. La N-acétyl-cystéine est un précurseur du glutathion qui est utilisé en clinique pour diverses indications, bien tolérée même à des doses élevées. Nous proposons donc une étude en double aveugle et cross-over dont le but est d'étudier si la N-acétyl-cystéine (à la dose de 2g/jour par voie orale) entraîne d'une part une élévation du glutathion cérébral (mesuré en résonance magnétique spectroscopique) et d'autre part une amélioration de l'état des patients (déterminé par des appréciations cliniques, psychopathologiques, neuropsychologiques, biochimiques et physiologiques), tout en enregistrant d'éventuels effets secondaires. Dans une première étape, cette étude devrait comporter au moins trente patients et durer deux à trois ans. Le groupe de Lausanne a ainsi la possibilité de se joindre à une recherche similaire en cours en Australie, où le groupe du Prof. David Copolov, inspiré par nos travaux, a entrepris une telle étude. Il est important de souligner qu'il ne s'agit pas d'une étude de médicament suggérée par une industrie pharmaceutique, mais du fruit d'une recherche médicale de base.

2. DUREE DE L'ETUDE

- 24 à 36 mois (durée d'investigation pour un patient : 4 mois, extensible à 8 mois)

3. FINANCEMENT

- Hospices Cantonaux Vaudois/ CHUV
- Fonds National Suisse de la Recherche Scientifique (F. n° 31 –55.924.98)

4. INVESTIGATEURS

Responsables du projet:

PD Dr Kim Q. DO CUENOD, Cheffe de Section de Recherche, Laboratoire Universitaire de Neurosciences Psychiatriques (LUNEP), DUPA

Prof. Michel CUENOD, Prof. invité, Faculté de Médecine, Université de Lausanne

Investigateur principal:

Dr Pierre BOVET, médecin adjoint, Section «E. Minkowski», DUPA

Moniteur :

Dr Thierry BUCLIN, médecin associé, Division de Pharmacologie Clinique, CHUV

Collaborateurs :

Prof. Peter BOESIGER, Institut für Biomedizinische Technik, EPFZ et UNIZH
Dr Philippe CONUS, médecin associé, Section « E. Minkowski » (DUPA)
Patricia DEPPEN, psychologue, LUNEP (DUPA)
Marie-Louise MATTHEY, psychologue, UREP (DUPA)
Prof. Reto MEULI, Service de radiodiagnostic, CHUV
Dr Dieter MEIER, Institut für Biomedizinische Technik, EPFZ et UNIZH
Jacqueline PICTET, doctorante, EPFL
Dr Martin PREISIG, médecin associé, Section Troubles Anxieux et de l'Humeur et UREP (DUPA)
Sylviane REYMOND, technicienne, LUNEP (DUPA)
Dr Alessandra SOLIDA, médecin- assistant, Section « E. Minkowski » et LUNEP (DUPA)
Dr Andreas TRABESINGER, Institut für Biomedizinische Technik, EPFZ et UNIZH
Prof. Jacques VAN DER KLINK, Département de Physique, EPFL
L'équipe soignante de la Section « E. Minkowski » participe au recrutement et à la surveillance des sujets.

Collaborateurs externes:

Prof. David COPOLOV, Directeur du Mental Health Research Institute of Victoria, Australie,
(http:// www.mhri.edu.au)

<u>Lieu de l'étude:</u>

Section «E. Minkowski », Sévelin 18, Lausanne; Hôpital de Cery, Prilly.
Laboratoire Universitaire de Recherche en Neurosciences Psychiatriques (LUNEP).

5. ETAT ACTUEL DE LA RECHERCHE

Théories actuelles : Deux hypothèses dominent actuellement la recherche biochimique sur la schizophrénie, reposant sur des troubles soit de la dopamine, soit du glutamate. Les évidences pour la théorie mettant en jeu une hyperactivité dopaminergique, sont l'induction d'un syndrome psychotique par les agonistes des récepteurs dopaminergiques, et les effets thérapeutiques favorables de leurs antagonistes. L'hypothèse d'une hypofonction glutamatergique, elle, est basée sur le fait que la phencyclidine, qui induit un syndrome psychotique, est un antagoniste du récepteur glutamatergique NMDA.

Ces hypothèses ont pourtant des limitations: les deux modèles expliquent en effet seulement une partie du spectre complexe de la symptomatologie schizophrénique, essentiellement les symptômes dits positifs (tels que délire et hallucinations), laissant inexpliquée la partie dite négative, souvent prédominante dans la phase résiduelle du trouble et responsable d'une bonne partie du handicap social engendré par la maladie. Ces théories ne fournissent pas non plus une explication satisfaisante des « symptômes de base » caractéristiques de la phase prodromique de la maladie ni, de façon générale, des manifestations de « trait » de la maladie.

Déficit en glutathion dans la schizophrénie : dans une étude du liquide céphalo-rachidien (LCR) de 26 schizophrènes non traités, parmi les 27 substances analysées, nous avons observé, par rapport à des contrôles, une diminution significative (de 27%) de la concentration de glutathion (GSH) (Do et al., 2000) et de son métabolite la γ-glutamylglutamine (de 16%) (Do et al., 1995). De plus, grâce à une méthode récemment développée en résonance magnétique spectroscopique (RMS) par le groupe du Prof. P. Boesiger de Zurich, le niveau de glutathion chez 18 patients s'est révélé être abaissé de 51% dans le cortex préfrontal médian (Do et al., 2000), une aire cérébrale dont les fonctions sont affectées dans la schizophrénie. L'évaluation sur le LCR et la RMS a été effectuée sur des patients en grande

partie non traités par des neuroleptiques. Cet élément fait supposer que le déficit en glutathion qui a été observé n'est en tout cas pas consécutif à la médication.

Hypothèse : déficit en glutathion comme facteur de vulnérabilité de la schizophrénie : le glutathion joue un rôle primordial dans la protection des cellules contre les effets toxiques des radicaux libres, produits entre autres par le métabolisme de la dopamine. Un déficit en glutathion tel que nous l'avons observé pourrait donc permettre à des processus dégénératifs de se développer dans l'environnement des terminaisons dopaminergiques, entraînant des microlésions du complexe associant terminaisons synaptiques et épines dendritiques. Ces lésions entraîneraient un déficit de la connectivité corticale qui pourrait être à l'origine des troubles cognitifs de la maladie. Le glutathion a également pour effet d'augmenter la réponse au glutamate du récepteur NMDA. Cet effet devrait être diminué si le glutathion est déficitaire, avec des conséquences analogues à l'application de phencyclidine, qui est un inhibiteur du récepteur NMDA et induit un syndrome psychotique. Nous proposons donc une hypothèse sur la pathophysiologie de la schizophrénie basée sur un déficit en glutathion comme facteur de vulnérabilité, hypothèse qui pourrait intégrer plusieurs aspects biologiques connus de la maladie. Cette hypothèse peut également rendre compte des troubles prémorbides, prodromiques et résiduels. L'effet nuisible d'un déficit en glutathion sur les terminaisons dopaminergiques pourrait être aussi compatible avec l'hypothèse d'un trouble neurodéveloppemental de la connectivité cortico-corticale et thalamo-corticale (Parnas et al., 1996) qui serait à la base des manifestations de trait. En effet, un déficit en glutathion au cours du développement pourrait entraîner une diminution progressive des contacts synaptiques et donc de la connectivité. Vers la fin de l'adolescence, l'augmentation de l'innervation dopaminergique combinée avec des stress psycho-sociaux, qui sont connus pour favoriser la libération excessive de dopamine, expliquerait la première décompensation psychotique.

Modèles de déficit en glutathion. Au cours des dernières années, notre laboratoire a développé des modèles *in vitro* et *in vivo* soumis à un déficit en glutathion, avec ou sans excès de dopamine, afin de tester si, dans ces conditions, il se produit des altérations morphologiques ou comportementales rappelant celles observées chez les patients atteints de schizophrénie (Do et al., in press).

(a) Dans des cultures de neurones, l'abaissement du glutathion associé à une adjonction de dopamine induit une diminution du nombre de processus dendritiques (analogues aux épines dendritiques, dont des études *post-mortem* ont montré qu'elles sont en nombre réduit dans les neurones du cortex préfrontal de schizophrènes) (Grima et al., 2003).

(b) Sur des tranches d'hippocampe, l'abaissement du glutathion entraîne des perturbations de la plasticité synaptique: le phénomène de « potentiation à long terme » ne peut plus être induit, ce qui est compatible avec une activation insuffisante du récepteur NMDA (Steullet et al., 2003).

(c) Chez le rat dont le glutathion a été abaissé de 50% pendant le développement (du 5eme au 24eme jour) et dont le taux de dopamine a été élevé par un bloqueur de sa recapture, on observe des anomalies des épines dendritiques susceptibles de conduire à des troubles de la connectivité ; cela évoque les déficits en épines dendritiques observés sur les pyramides préfrontales de schizophrènes (Gheorghita et al, 2003 ; Castagne et al., 2002, 2003; Rougemont et al., 2003, Castagne et al., in press (a) et (b)).

(d) Dans ces mêmes conditions, l'immunoréactivité à la parvalbumine est fortement réduite dans le cortex cingulaire antérieur, suggérant une anomalie des neurones gabaergiques (telle qu'elle a été décrite dans le cerveau de schizophrènes) (Cabungcal et al., observations non publiées).

(e) Chez le rat dont le glutathion a été abaissé de 50% pendant le développement seulement (du 5eme au 16eme jour), on observe, chez l'animal adulte, un important déficit de la mémoire des objets tant visuels qu'olfactifs. Ce trouble comportemental n'est pas sans rappeler certains déficits cognitifs observés dans la schizophrénie (Cabungcal et al., 2003).

Résultats préliminaires de l'étude « Schizophrénie, troubles apparentés et glutathion » (approbation de la Commission d'Ethique du 19/07/99) sur du matériel provenant de patients (sang, biopsie de peau) : des observations récentes (non publiées) révèlent des altérations des acides aminés soufrés et des enzymes impliquées dans le métabolisme du glutathion. Fait particulièrement intéressant, l'anomalie d'une de ces enzymes est en corrélation avec la symptomatologie : plus elle est basse, plus les symptômes sont marqués.

6. MEDICAMENT DE L'ETUDE

L'ensemble de ces résultats renforce la validité de l'hypothèse proposée et justifie un essai clinique avec la N-acétyl-cystéine (NAC), un médicament connu pour élever le niveau du glutathion dans les tissus, afin de renforcer tant son action anti-oxydante que son effet sur le récepteur NMDA. Il est important de souligner qu'il ne s'agit pas ici d'une étude de médicament proposée par une industrie pharmaceutique, mais du fruit d'une recherche médicale de base.

La NAC est un dérivé de la cystéine avec un group thiol libre, utilisé en clinique pour ses propriétés anti-oxydantes depuis les années 50. Introduite pour les affections respiratoires chroniques, la NAC a ensuite été approuvée comme antidote pour l'intoxication au paracétamol. Plus récemment, la NAC a été l'objet de nombreuses études pour ses effets biologiques, incluant des applications cliniques potentielles.

La NAC a comme effet de réduire la concentration des radicaux libres et d'autres oxydants. Son action anti-oxydante se fait d'une part par l'inactivation directe des espèces oxygénées réactives grâce au groupe thiol libre de la molécule et la formation de NAC- disulfide comme produit final. D'autre part, la NAC exerce son action anti-oxydante surtout en induisant la biosynthèse du GSH in vivo. Un des métabolites de la NAC est en fait la cystéine qui est un précurseur essentiel de la synthèse du glutathion. La disponibilité en cystéine est un facteur crucial pour la réserve cérébrale de glutathion puisque ce dernier passe difficilement la barrière hémato-encéphalique. La NAC joue également un rôle important dans l'expression des gènes liés au stress oxydatif, à travers son effet sur les facteurs de transcription tels que NF-κB et AP1 (Cotgreave 1997).

La NAC est un médicament commercialisé (Fluimucil®, Solmucol® et autres) et approuvé en qualité de mucolytique pour le traitement des bronchites et en qualité d'antidote spécifique lors d'intoxication aiguë au paracétamol. Sa résorption après prise orale est rapide et complète. La bio-disponibilité de la NAC est relativement limitée après administration orale en raison de sa désacétylation en cystéine au niveau de la paroi intestinale, des hépatocytes et des cellules endothéliales. Après une administration orale de 600 mg, sa demi-vie d'élimination est d'environ 2,15 heures. Le 30% de la dose est éliminé par voie rénale directe. Les métabolites principaux sont la cystéine et la cystine, ainsi que de petites quantités de taurine et des sulfates.

Parmi les contre-indications connues figurent l'ulcère peptique actif et l'hypersensibilité connue à la molécule. On observe rarement de légers troubles gastro-intestinaux tels que brûlures d'estomac, nausée, vomissement ou diarrhée et dans des cas exceptionnels de l'urticaire, des maux de tête et de la fièvre. Les réactions anaphylactiques n'ont été observées que lors d'administration intraveineuse à dose élevée. La posologie recommandée habituellement en clinique comme mucolytique est de 600 mg/j pour les adultes, toutefois l'administration de NAC est bien tolérée jusqu'à 5 g/j (Louwerse et al. 1995). La dose de 2 g/j produit un taux plasmatique élevé et persistant de cystéine.

Copolov et al. (communication personnelle) ont effectué un essai clinique contrôlé en double aveugle visant à établir l'efficacité et la tolérance de la NAC (2 g/j) dans un groupe de sujets schizophrènes. Les observations préliminaires de l'étude (encore en aveugle) laissent espérer un effet favorable de la NAC sur la symptomatologie schizophrénique négative et positive.

Concrètement, nous prévoyons d'utiliser pour cette étude des gélules de NAC dosées à 500 mg. Ces gélules seront spécialement préparées par un pharmacien, et conditionnées en flacons de 250 gélules. Des gélules exactement semblables remplies de lactose serviront de placebo. Afin que le double aveugle puisse être respecté malgré l'odeur sulfureuse caractéristique de la NAC, il est prévu de parfumer l'intérieur des flacons à l'aide d'un composé odorant de couverture. L'étiquette des flacons comportera le numéro d'inclusion de chaque patient et la mention « première période » ou « deuxième période ». NAC et placebo seront assignés aléatoirement à la première ou à la deuxième période selon une liste de randomisation détenue uniquement par le pharmacien. Des enveloppes individuelles scellées contenant la séquence de chaque patient inclus seront confiées au médecin investigateur principal, le Dr P. Bovet.

Vu l'absence d'un effet connu de la NAC comme antagoniste dopaminergique, nous ne nous attendons pas à ce que ce médicament induise ou aggrave les effets extrapyramidaux secondaires chez les patients sous traitement neuroleptique. On pourrait même imaginer que la NAC puisse améliorer les symptômes extrapyramidaux dus aux neuroleptiques grâce à son action anti-oxydante. De nombreux auteurs (Cadet et al. 1986; Reddy et al. 1999, Yao et al. 2001) en effet ont proposé que des mécanismes de stress oxydatif interviennent dans la physiopathologie de certains des effets secondaires, comme les dyskinésies tardives.

7. OBJECTIFS DE L'ETUDE

Notre étude vise à évaluer si l'administration orale de NAC à des patients schizophrènes stabilisés sous traitement neuroleptique permet de reconstituer les réserves physiologiques de glutathion intracérébral, améliorant ainsi la symptomatologie des troubles du spectre de la schizophrénie. L'observation d'une amélioration de ces symptômes représenterait un élément en faveur de notre hypothèse qu'un déficit en glutathion constitue un facteur de vulnérabilité envers la schizophrénie.
Cette étude vise à répondre aux questions suivantes:

(a) L'administration de NAC peut-elle augmenter la concentration intracérébrale de GSH chez les patients schizophrènes ? L'évaluation de la concentration de GSH sera effectuée grâce à un examen en RMS, qui est un procédé non-invasif.

(b) L'administration de NAC est-elle accompagnée par une efficacité clinique mesurable en termes d'amélioration des symptômes schizophréniques (symptômes positifs et négatifs, déficits cognitifs, symptômes de base) ? L'évaluation des ces variables sera effectuée à l'aide

d'instruments de psychopathologie et d'une batterie neuropsychologique spécifique pour les troubles du spectre.

(c) Y-a-t-il parmi les effets cliniques une modification des effets secondaires du traitement neuroleptique pris par les patients durant l'étude ? Les variations des effets secondaires des neuroleptiques, notamment les effets dits "extrapyramidaux", seront également évaluées afin d'établir une relation entre l'action antioxydante de la NAC et son rôle protecteur potentiel contre ces effets secondaires.

8. METHODOLOGIE : PLAN DE L'ETUDE, SUJETS ET INVESTIGATIONS

Afin de vérifier l'hypothèse de cette étude, notre objectif est qu'un minimum de 15 patients terminent les 4 mois d'étude. Estimant un taux d'abandon pouvant s'élever jusqu'a 50% des patients inclus, nous envisageons de recruter environ trente patients, autant que possible sur une année.

Après l'inclusion dans l'étude, les patients seront randomisés en deux groupes: un groupe qui recevra le médicament de l'étude (2 capsules de 500 mg de NAC 2 fois/j pour une dose totale de 2 g/j) et un groupe qui recevra le placebo durant 2 mois. Après les deux premiers mois d'étude, nous procéderons au cross-over des deux groupes pour une nouvelle période de 2 mois (cf. section *Durée de l'étude*).

Pourront être inclus: les patients avec un diagnostic de Schizophrénie, de Trouble schizophréniforme ou de Trouble schizotypique (selon les critères du DSM IV). Le diagnostic clinique sera vérifié par l'un des médecins-cadre. Seront exclus: a) les patients dont la symptomatologie psychotique ne permet pas, momentanément en tout cas, une collaboration suffisante; b) les patients présentant une co-morbidité (dépendance à l'alcool, abus de drogues, affection somatique majeure); c) les patients ayant une atteinte organique cérébrale, anamnestique ou actuelle, documentée (cf. section *Sélection des sujets*).

Le recrutement des patients sera effectué au sein de la population ambulatoire de l'unité du DUPA spécialisée dans les troubles du spectre de la schizophrénie, la Section "E. Minkowski". Vu que plusieurs des patients de cette Section ont participé à l'étude sur le glutathion dans les troubles du spectre de la schizophrénie, nous envisageons de proposer aux patients ayant participé à la première étude, la possibilité de participer à cette deuxième investigation s'ils le désirent. Les patients seront sélectionnés avec l'accord du médecin en charge de leur suivi. L'information sera présentée aux sujets par l'un des investigateurs ou des collaborateurs à cette étude (Dr P. Bovet, Dr. P. Conus, P. Deppen, Dresse A. Solida).

Le recrutement pour cette étude concernera des patients stabilisés, présentant des symptômes psychotiques positifs et/ou négatifs résiduels, stables depuis au moins 3 mois. Les patients avec un diagnostic de trouble schizotypique, ne présentant donc pas de symptômes psychotiques florides, pourront également être inclus après passation de l'autoquestionnaire SPQ.

Le traitement neuroleptique, ni non plus un éventuel changement de la posologie pendant l'étude ne constituent un critère d'exclusion. Le remplacement d'un neuroleptique par un autre constituera par contre un critère de retrait de l'étude. L'association d'un autre neuroleptique à celui préexistant sera acceptée, pourvu qu'il s'agisse d'une association au traitement primaire. L'utilisation d'autres médicaments psychotropes sera acceptée si le patient est sous cette médication depuis au moins un mois. Les patients sous thymorégulateurs (lithium, valproate, topiramate, lamotrigine et carbamazepine) ne seront pas inclus.

Pendant l'étude, les patients effectueront des examens psychopathologiques, neuropsychologiques, biochimiques, neurophysiologiques (EEG, potentiels évoqués) et une résonance magnétique spectroscopique. La BATTERIE A (comprenant les échelles d'évaluation neurologique et psychopathologique, exception faite du QPF) sera passée à quinzaine. La BATTERIE B (tests neuropsychologiques, QPF, examens biochimiques, RMS et examens neurophysiologiques) une fois tous les 2 mois, à savoir au début, à deux mois et à la fin de l'étude de base (cf. section *Méthodes d'investigation*). Si un sujet doit interrompre l'étude de façon prématurée, la batterie A sera passée au moment de l'interruption. La liste complète et les caractéristiques de chaque examen sont présentées en détail à la section *Schéma général de l'étude* ainsi qu'à la section *Méthodes d'investigation*.

➢ **Durée de l'étude**

- La durée de l'étude est fixée 4 mois, extensible à 8 mois. Durant les 4 premiers mois, 9 visites (V1 à V9) seront réparties de la façon suivante (cf. *Schéma général de l'étude*): I - II: après randomisation (groupe NAC ou placebo), les tests de BATTERIE A seront effetués toutes les 2 semaines (V2,V3, V4). Une évaluation plus large, comprenant les BATTERIES A + B, sera effectuée au début de l'étude (V1) et à la fin des 2 mois (V5).
- III – IV: à la fin des deux premiers mois, le cross-over des deux groupes de patients sera effectué (V5). L'étude sera poursuivie de manière analogue à la période précédente avec des évaluations toutes les 2 semaines (V6, V7, V8) et une évaluation plus large à la fin des 4 mois (V9).

  Notons que la durée de passation des BATTERIE A + B est d'environ 5h (pour V1, V5 et V9) et la durée de la BATTERIE A seule est de 1h45 (pour V2-V4 et V6-V8). Chaque

évaluation peut être répartie sur plusieurs séances.

- V-VIII: si le patient souhaite continuer, l'étude pourra se poursuivre encore 4 mois. Dans ce cas, nous envisageons de décoder et de continuer en ouvert. Un document de décision sera établi, fixant les critères retenus avant l'ouverture du code. Le placebo sera interrompu si c'était le traitement le plus efficace. En revanche, si c'est la NAC qui est la plus efficace, son administration sera poursuivie. La BATTERIE A sera répétée à la fin du 6e mois et au 8e mois, à la fin de l'étude. En outre, un examen physique ainsi qu'une révision des effets secondaires et de la compliance seront effectués tous les mois (V10,V11, V12, V13).

➢ **Sélection des sujets**

♦ Critères d'inclusion

- Sujets (hommes et femmes) francophones âgés de 18 à 65 ans.
- Diagnostic de schizophrénie, trouble schizophréniforme ou trouble schizotypique selon les critères DSM-IV.
- Diagnostic de trouble schizotypique selon les critères DSM-IV et confirmation auto-questionnaire SPQ (Dumas et al.2000)

♦ Critères d'exclusion

- Grossesse en cours ou programmée durant la période de l'étude. A ce titre, un test de grossesse sera effectué au moment de l'entrée dans l'étude, à 2 mois et à la fin de l' essai. En outre, les investigateurs s'assureront que la participante utilise un moyen de contraception efficace pendant toute la durée de l'étude.
- Changement de neuroleptique (l'augmentation de la posologie ou l'association d'un deuxième neuroleptique au neuroleptique "primaire" seront tolérées)
- Comorbidités somatiques graves, comprenant entre autres: anamnèse d'ulcère peptique, pathologies inflammatoires chroniques, infectieuses (par ex. SIDA), immunitaires, tumorales, dysfonctions hépatiques, rénales ou/et thyroïdiennes ainsi que les atteintes organiques du SNC.
- Diagnostic de dépendance à une substance selon les critères DSM IV.
- Trouble psychotique induit par une substance
- QI inférieur à 70
- Allergie connue à la N-acétylcystéine
- Traitement avec d'autres antioxydants (Vit. E, sélénium, complexes polyvitaminés)

Schéma général de l'étude

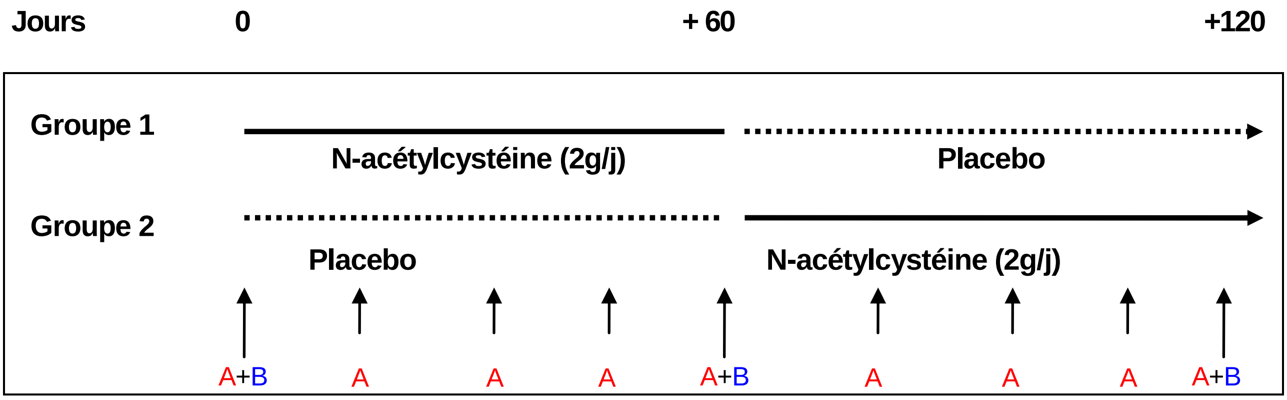

		V1 (J 1)	V2 (J15)	V3 (J30)	V4 (J45)	V5 (J60)	V6 (J75)	V7 (J90)	V8 (J105)	V9 (J120)
	Critères inclusion/exclusion	+								
	Signes vitaux	+	+	+	+	+	+	+	+	+
	Ex. Physique (+ test grossesse)	+				+				+
	Effets indésirables		+	+	+	+	+	+	+	+
	Evaluation compliance		+	+	+	+	+	+	+	+
A	AIMS	+	+	+	+	+	+	+	+	+
	Echelle de S-Angus	+	+	+	+	+	+	+	+	+
	Echelle de Barnes	+	+	+	+	+	+	+	+	+
	PANSS	+	+	+	+	+	+	+	+	+
	CGI	+	+	+	+	+	+	+	+	+
	GAF	+	+	+	+	+	+	+	+	+
B	QPF	+				+				+
	Tests neuropsychologiques	+				+				+
	Ex. de laboratoire	+				+				+
	RMS	+				+				+
	EEG/Potentiels évoqués	+				+				+

➢ Méthodes d'investigation

**BATTERIE A**	**1. Echelles neurologiques**<br>• **AIMS**<br>• **Echelle de Simpson- Angus**<br>• **Echelle de Barnes**<br>**2. PANSS**<br>**3. CGI**<br>**4. GAF**
**BATTERIE B**	**1. QPF**<br>**2. Tests neuropsychologiques**<br>**3. Examens de laboratoire**<br>**4. RMS**<br>**5. EEG/Potentiels évoqués**

- **Examen physique et signes vitaux**

Un examen physique sera effectué à l'entrée de l'étude (V1), à 2 mois (V5) et à la fin (V9) ainsi que, le cas échéant, aux mois VI et VIII de l'étude. Le contrôle de la tension artérielle, de la température et du poids seront effectués à chaque visite (V1- V9). Pour les femmes qui participent à l'étude, un test de grossesse est prévu à l'entrée de l'étude (V1), à 2 mois (V5) et à la fin (V9).

- **Effets indésirables et compliance:**

La révision avec les patients des effets secondaires possibles et/ou d'autres événements significatifs sur le plan de la santé sera effectuée à chaque visite. Le médicament de l'étude sera donné avec un semainier tous les 15 jours. Le contrôle de la compliance sera limité au décompte des capsules restantes dans le semainier.

- **<u>Echelles neurologiques d'évaluation des effets extrapyramidaux</u> (AIMS, échelle de Simpson-Angus et échelle de Barnes, BATTERIE A)**

Il s'agit d'échelles qui permettent l'évaluation standardisée de la symptomatologie extrapyramidale. Ces échelles permettent l'appréciation de l'extension et de la sévérité des effets secondaires extrapyramidaux grâce à un examen clinique relativement simple.

L' AIMS (Abnormal Involuntary Movements Scale) est une échelle introduite par le National Institute of Mental Health afin de relever quantitativement les hypercynésies secondaires (tremblement exclu) au traitement neuroleptique.

L'échelle de Simpson-Angus est un outil simple et bien standardisé, sensible à d'autres effets extrapyramidaux (tels que le tremblement et d'autres signes parkinsoniens comme la rigidité et l'akinésie).

L'échelle de Barnes permet l'évaluation spécifique de l'akathisie, autre effet secondaire possible du traitement neuroleptique, caractérisée par une symptomatologie motrice et psychique perçue comme désagréable par le patient. Pour cette raison, cette échelle combine une évaluation clinique objective et l'auto-appréciation du patient.

- **<u>Evaluation psychopathologique</u>**

  - ***L'échelle de symptomatologie positive et négative (Positive and Negative Syndrome Scale (PANSS, BATTERIE A)***

  Cette échelle permet d'évaluer la sévérité de symptômes psychotiques positifs et négatifs de la schizophrénie. Il s'agit d'un entretien semi-structuré de 45 minutes environ.

  - ***Le questionnaire des plaintes de Francfort (Frankfurter Beschwerde Fragebogen – QPF, BATTERIE B)***

  Il s'agit d'un auto-questionnaire portant sur des troubles subjectifs, appelés "symptômes de base". Il s'agit de troubles perceptifs, cognitifs discrets ou moteurs qui sont fréquents dans les phases prodromiques et intercurrentes de la schizophrénie, et que l'on trouve aussi chez les personnes au profil schizotypique. Ce questionnaire est informatisé et sa passation dure une vingtaine de minutes.

  - ***L'échelle d'évaluation globale du fonctionnement (Global Assessment of Functioning – GAF, BATTERIE A).***

  Cette échelle est particulièrement indiquée pour suivre globalement les progrès cliniques du patient; elle permet d'estimer le fonctionnement actuel du sujet au moyen d'une note unique. Il s'agit pour le

clinicien d'évaluer le fonctionnement psychologique, social et professionnel sur un continuum hypothétique allant de la santé à la maladie (entre 100 et 0).

- ***L'échelle d'impression clinique globale (Clinical Global Impression – CGI, BATTERIE A)***

La CGI donne une idée générale de l'état du patient, de même que du rapport bénéfice/ risque des psychotropes utilisés. Il s'agit de trois rubriques permettant l'évaluation ponctuelle de la gravité de la maladie, de l'amélioration globale ainsi que de l'effet du médicament (efficacité thérapeutique et effets secondaires). Cette échelle, de même que la GAF, est remplie par le clinicien seul en quelques minutes.

- **Evaluation neuropsychologique (BATTERIE B)**

Une batterie de tests neuropsychologiques a été développée afin d'investiguer, sur le plan fonctionnel, des particularités cognitives de la population étudiée. Cette batterie, en association avec trois sous-tests de la WAIS, permet principalement de tester certaines fonctions frontales (mémoire de travail, planification, certaines composantes de l'attention), qui sont des fonctions particulièrement déficitaires chez les patients schizophrènes, ainsi que la perception visuelle. Ces tests sont donc bien adaptés à une étude de corrélation avec les données issues de la RMS, qui investigueront les régions pré-frontale et occipitale.

La batterie de tests neuropsychologiques est montée sur ordinateur, et elle est à disposition des membres de la Commission (disquettes PC en annexe). Quant aux sous-tests de la WAIS-III pris en compte dans cette évaluation, il s'agit des trois épreuves qui examinent l'indice de mémoire de travail. La passation de ces trois sous-tests et de la batterie informatisée dure environ une heure et demie (fractionnement possible).

- **Examens de laboratoire (BATTERIE B)**

- ***Prise de sang***

La Dresse A. Solida sera chargée des prélèvements sanguins (25 ml). Les analyses suivantes seront effectuées :

I. dosage du taux plasmatique de glutathion et des ses métabolites
II. analyse des activités enzymatiques liées au glutathion et à d'autres systèmes antioxydants
III. analyse génétique des enzymes corrélées au métabolisme du glutathion
IV. formule sanguine et tests des fonctions hépatique, rénale et thyroïdienne

Le formulaire de consentement dont l'acceptation par le patient est le critère préalable à sa participation à l'étude, mentionnera spécifiquement l'analyse génétique citée au point III et le fait que

les échantillons soient détruits une fois l'analyse terminée ou immédiatement à la demande expresse du patient.

- ***Prélèvement d'urine***

Les résultats obtenus à ce jour permettent de suspecter un déficit enzymatique chez les patients. Un tel déficit pourrait conduire à l'accumulation de certaines substances éliminées par le rein (5-oxoproline). C'est une piste qui nous semble très prometteuse, par les perspectives pratiques qui pourraient en découler (dépistage non invasif). Les désagréments pour les sujets participant à l'étude sont minimes : il s'agit d'un prélèvement d'urine tout à fait ordinaire, qui s'effectuerait juste avant la prise de sang.

- **Résonance Magnétique Spectroscopique (BATTERIE B)**

Une étude préliminaire de Do et al. (2000) a observé une diminution significative du taux de glutathion au niveau du cortex préfrontal chez un groupe de 18 schizophrènes. La concentration de glutathion dans des aires différentes du cerveau sera donc évaluée, avec une attention particulière pour la région préfrontale/temporale afin d'analyser des variations inter- ou intra-individuelles chez les patients traités avec NAC ou placebo.

L'étude se fait d'une manière non invasive avec un tomographe de résonance magnétique nucléaire pour le corps entier. L'appareil de MRS ne dégage pas de radioactivité, mais génère un champ magnétique. Avant l'analyse, le sujet recevra une feuille d'information sur l'étude en RMS ainsi qu'un questionnaire (annexes) qu'il devra remplir et signer. Le procédé, qui est semblable à un examen neuroradiologique en résonance magnétique, sera expliqué au patient. Pour l'examen, le sujet, allongé confortablement sur un lit mobile, sera introduit dans l'aimant. Le contact avec l'accompagnateur et le personnel RMS est possible pendant toute la durée de l'examen. La tête est fixée dans un cadre spécial afin d'éliminer les artefacts provenant des mouvements pendant l'enregistrement. Le cas échéant, l'examen pourrait être arrêté à tout instant à la demande du sujet. La durée totale de l'examen est d'environ 60 minutes.

La méthode n'étant pas encore définitivement établie dans le département de Radiologie du CHUV sur un appareil Siemens de 1,5 T, cet examen sera effectué à Zurich dans l'Institut für Biomedizinische Technik (Prof. Peter Bösiger, EPFZ et UNIZH). C'est là que la méthode de détermination du glutathion a été développée en 1998 à notre demande (Trabesinger et al., 1999) et utilisée dans notre première étude (Do et al., 2000). La validité de cette méthode a été confirmée aux USA par Gruetter et collaborateurs (in press). Elle fonctionne actuellement à l'Institut für Biomedizinische Technik avec un appareil Philips d'une puissance de 3 T, qui garantit une meilleure résolution des signaux et un

meilleur rapport des signaux sur le bruit de fond. La mesure, qui exige une surveillance constante par un physicien spécialisé, sera faite en principe par les mêmes personnes qui ont opéré les mesures de la première étude. Bien qu'elle soit assez lourde, cette solution offre donc toutes les garanties de qualité et de fiabilité souhaitable pour une mesure indispensable à la validité de l'étude. Le déplacement des patients nous paraît toutefois tout à fait envisagable, étant donné que l'examen en MRS n'a lieu qu'à trois reprises au cours de l'étude. En outre, le transport et l'accompagnement des patients seront assurés par nos soins. Dans une phase ultérieure de l'étude, si la méthode fonctionne efficacement au CHUV, les déterminations du glutathion en MRS seront reprises à Lausanne.

- **EEG/Potentiels évoqués (BATTERIE B)**

Cette partie de l'étude se ferait en collaboration avec le Prof. R. Meuli, les Drs P. Maeder et M. Knyazeva du Service de Neuroradiologie du CHUV. La P300 est une onde qui apparaît à l'électro-encéphalogramme 300 millisecondes après l'exposition à un stimulus (le plus classiquement, un son d'une certaine fréquence que le sujet doit identifier, ce qu'on appelle « auditory oddball paradigm »). Des anomalies de l'amplitude ou du délai d'apparition de l'onde P300 ont été constatées à plusieurs reprises chez les patients souffrant de schizophrénie, et ont pu être corrélées avec la gravité de certains symptômes, notamment la symptomatologie négative (Mathalon et al., 2000). A l'heure actuelle, on peut dire que les anomalies de la P300 sont parmi les marqueurs électrophysiologiques les plus fiables que l'on connaisse. Nous souhaitons mettre en rapport les variations intra- et interindividuelles des paramètres cliniques, des résultats biologiques et de la mesure de la P300 chez les sujets participant à cette étude.

Par ailleurs, il a été décrit chez les patients souffrant de schizophrénie une désynchronisation de l'activité électrique spontanée (le sujet étant « au repos ») mesurée par EEG de surface. Cette anomalie pourrait être le corrélat électro-physiologique d'un défaut de la connectivité cérébrale, concept central dans l'hypothèse que nous avons formulée d'un déficit en glutathion dans la schizophrénie. La mesure des potentiels évoqués sera donc complétée par un enregistrement de trois minutes en condition de repos afin d'évaluer ce paramètre.

L'examen se déroulerait au CHUV sur une durée de 60 minutes environ une fois le sujet installé. L'enregistrement de l'activité cérébrale électrique se déroule en 2 phases :

I. Mesure de la connectivité sans stimulation externe durant 3 minutes (yeux fermés).
II. Mesure des potentiels évoqués : auditory oddball paradigm.

L'examen n'est pas invasif, n'est pas douloureux et ne présente aucun danger.

9. QUESTIONS ETHIQUES

La prise en charge du sujet ne sera pas modifiée par l'étude en cours. Le patient poursuivra son traitement médicamenteux habituel dont la posologie pourra être modifiée en tout temps selon l'avis du médecin traitant.

Bien qu'il soit préférable pour le succès de l'étude que les sujets acceptent l'ensemble des examens, il leur sera dit clairement qu'ils sont libres de refuser de participer à l'un ou l'autre de ces examens. Avant que le sujet ne soit recruté pour l'étude, il doit recevoir (si nécessaire son représentant légal) une explication complète orale et écrite (annexe) sur le but et le plan de l'étude. Un formulaire de consentement (annexe) doit être signé par le sujet, le cas échéant également par son représentant légal, dans lequel il est clairement expliqué qu'il lui est possible d'arrêter l'examen sans en subir de désavantages personnels.

La survenue d'éventuels effets secondaires de la NAC sera contrôlée tout au long de l'étude. Le sujet pourra en tout temps être exclu de l'étude à sa demande ou dans le cas où les effets secondaires devraient être jugés préoccupants.

Le prélèvement sanguin sera effectué avec toutes les précautions nécessaires.

Les évaluations psychopathologiques et neuropsychologiques envisagées présentent une utilité directe pour la prise en charge des patients et en font souvent partie intégrante. Les patients continueront bien entendu à être suivis régulièrement par leur psychiatre habituel. En cas de besoin, ils pourront s'adresser au médecin en charge de l'étude, la Dresse A. Solida.

Les effets indésirables de l'examen en RMS pour le sujet pourraient résulter d'une claustrophobie, de l'impossibilité de rester immobile pendant plus de 30 minutes ou de la présence d'implants magnétiques dans le corps ou de corps étrangers non magnétique dans la région de la tête. Cependant ces facteurs prédisposants pourront être exclus lors du questionnaire (annexes) avant l'examen. Le sujet concerné ne subira pas l'examen RMS, tout en poursuivant l'étude. D'autres risques provenant de ce type d'examen ne sont pas connus. Dans le cas de la détection accidentelle d'une lésion structurelle du SNC (tumeur, anévrisme), le radiologue en informera le médecin responsable du projet (le Dr P. Bovet), qui informera à son tour le patient ou son médecin traitant de la nécessité éventuelle d'investigations ultérieures (la RMS ne permet pas la visualisation claire de la structure du cerveau, ayant été conçue comme technique pour visualiser l'activité métabolique du cerveau: une lésion pourra donc être suspectée mais pas définie avec certitude).

A travers l'évaluation psychopathologique, de même qu'à travers les tests

neuropsychologiques, le sujet peut être confronté à ses difficultés personnelles ainsi qu'à ses limites. De plus, ces évaluations requièrent d'importants efforts de concentration et certaines questions peuvent être ressenties comme intrusives. Cependant, ces examens seront réalisés par un clinicien expérimenté (psychiatre, psychologue), spécialement formé à ces techniques d'évaluation. Celui-ci sera attentif à encadrer le sujet lors de la passation des différentes échelles, de façon à ce que celle-ci se déroule au mieux. En outre, comme mentionné précédemment, l'analyse fine des difficultés du patient que permettent ces évaluations constitue un apport utile à sa prise en charge.

Toutes les précautions seront prises pour garantir la confidentialité absolue des données recueillies. Les cliniciens et toute personne travaillant dans le cadre de cette étude sont tenus au secret professionnel. Il sera attribué à chaque sujet un numéro d'identification. Ce numéro sera tiré au hasard, et non pas construit à partir d'éléments d'identité (comme le sont p.ex. les numéros AVS). Un fichier sera constitué sur un programme de type FileMaker, contenant les renseignements suivants: nom et prénom du sujet, sexe, date de naissance, adresse au moment de l'examen, numéro d'identification. Ce fichier sera protégé par un mot de passe connu exclusivement par le responsable clinique de l'étude, le Dr Pierre Bovet. Il sera constitué par et sur l'ordinateur du Dr P. Bovet. Une copie du fichier sera périodiquement adressée, à fins de sauvegarde, au chef du DUPA. Un autre fichier sera constitué au laboratoire de neurosciences. Il contiendra les résultats des investigations. Il s'agira de données cliniques (diagnostic, données sémiologiques, résultats des tests neuropsychologiques) et des données de laboratoire. Les seules données identitaires des patients qui y figureront seront: le numéro d'identification, le sexe, et l'année de naissance. La liste de randomisation sera conservée par le pharmacien du DUPA. Ni les cliniciens, ni les neuroscientistes ne pourront mettre en relation l'identité d'un sujet avec sa médication (NAC / placebo) ou les résultats des investigations, avant que celles-ci ne soient terminées, analysées et mises en perspective. Il ne sera donc pas possible de répondre à d'éventuelles demandes individuelles des sujets à propos des résultats biologiques. Par contre, il en sera autrement, si les sujets le souhaitent, pour les résultats des tests neuropsychologiques et du QPF, dans le sens où ceux-ci peuvent présenter une utilité directe pour le patient et sa prise en charge. Les sujets seront informés de ces limitations. Les risques sont couverts par l'assurance des Hospices.Cantonaux.

A titre de défraiement, les sujets se verront allouer la somme de Fr. 50.- au début de l'étude, 50.- au cross-over et 100.- à la fin.

10. BIBLIOGRAPHIE

American Psychiatric Association, (1994). Diagnostical and Statistical Manual of Mental Disorders , Fourth Edition. Washington, DC: American Psychiatric Association.

Barnes, T.R.E. (1989) A rating scale for drug-induced akathisia. British Journal of Psychiatry. 154: 672- 676.

Cabungcal JH, Castagné V, Hornung J-P, Cuénod M, Do KQ, Schenk F, Visual and olfactory place learning deficit in rats with low glutathione during development: a behaviour model with relevance to schizophrenia, Acta Neurobiologiae Experimentalis, 63, Suppl., 31, 2003

Cadet J.L., Lohr JB, Jeste DV (1986) Free radicals and tardive dyskinesia. Trends Neurosc 1986; 9: 107 – 108

Castagne V, Rougemeont M, Cuenod M, Do KQ (2002) Glutathione deficit in schizophrenia: development of an animal model. Schizophr.Res. 53:151

Castagne V., Rougemont M., Cuénod M., and Do K.Q (2003): A rat model with relevance to schizophrenia: Low brain glutathione levels during development induce long-term cognitive deficit, Schizophrenia Research, 60:105

Castagne V., Rougemont M., Cuénod M., and Do K.Q, Low brain glutathione and ascorbic acid associated with dopamine uptake inhibition during rat's development induce long-term cognitive deficit: relevance to schizophrenia, Neurobiology of disease, in press (a).

Castagne V., Cuenod M, Do KQ: An animal model with relevance to schizophrenia: sex-dependent cognitive deficits in rats induced by glutathione synthesis and dopamine uptake inhibition during development, Neuroscience, in press (b).

Cotgreave I.A. (1997) N-Acetylcysteine: pharmacological considerations and experimental and clinical applications. Advances in Pharmacology, 38 : 205-227

Do KQ, Lauer CJ, Schreiber W, Zollinger M, Gutteck Amsler U, Cuenod M, Holsboer F (1995) gamma-Glutamylglutamine and taurine concentrations are decreased in the cerebrospinal fluid of drug-naive patients with schizophrenic disorders. J.Neurochem. 65:2652-2662.

Do KQ, Trabesinger AH, Kirsten-Kruger M, Lauer CJ, Dydak U, Hell D, Holsboer F, Boesiger P, Cuenod M (2000) Schizophrenia: glutathione deficit in cerebrospinal fluid and prefrontal cortex in vivo. European Journal of Neuroscience 12:3721-3728.

Do K.Q.; Bovet P.; Castagné V.; Gheorghita F.; Hornung J-P.; Steullet P.; and Cuenod M., Glutathione deficit in schizophrenia: a new vulnerability factor for misconnectivity, Swiss Archives of Neurology and Psychiatry, in press

Dumas P., Bouafia S., Gutknecht C., Saoud M., Dalery J., d'Amato T. (2000) Validation of the French version of the Raine Schizotypal Personality Disorder questionnaire- categorial and dimensional approach to schizotypal personality traits in a normal student population. Encéphale; Sep-Oct; 26 (5). 23- 9.

Gheorghita F, Castagne V, Do KQ, Cuenod M, Hornung J-P, Glutathione deficit during postnatal development induces abnormal dendritic spine morphology in the rat prefrontal cortex, Journée de Recherche, CHUV, 2003

Grima G, Benz B, Parpura V, Cuenod M, Do KQ , Dopamine-induced oxidative stress in neurons with glutathione deficit: implication for schizophrenia. Schizophr.Res. 62, 213-224, 2003

Guy W. (1976) ECDEU Assessment Manual for Psychopharmacology. Washington, DC: Department of Health, Education and Welfare.

Kay, S.R., Opler, L.A. & Lindenmayer, J.P. (1986). The Positive and Negative Syndrome Scale (PANSS): rationale and standardization. Br. J. Psychiatry, 1989, 155, (suppl. 7), 59-65.

Mathalon DH, Ford JM, Pfefferbaum A (2000) Trait and state aspects of P300 amplitude reduction in schizophrenia: a retrospective longitudinal study. Biol.Psychiatry 47:434-449.

Parnas J, Bovet P, Innocenti GM (1996) Schizophrenic trait features, binding, and cortico-cortical connectivity: a neurodevelopmental pathogenetic hypothesis. Neurology, Psychiatry and Brain Research 4:185-196.

Reddy RD, Yao Jk. (1999) Membrane protective strategies in schizophrenia: conceptual and treatment issues. In : Peet M, Glen I, Horrobin DF, editors. Phospholipid spectrum disorders in psychiatry. Lancashire UK: Marius Press,: 75 – 88.

Rougemont M, Do KQ, Castagne V (2003) A new model of glutathione deficit during development: effect of glutathione deficit on lipid peroxidation in the rat brain. J.Neurosci.Res. 70:774-783.

Simpson G.M., Angus J.W.S. (1970) A rating scale for Extrapyramidal Side Effects. Acta Psychiatrica Scandin., 212: 11- 19.

Steullet, P., Cuénod, M., and Do, K.Q. (2003): Effect of glutathione deficit on neurotransmission and synaptic plasticity in rat hippocampus : relevance for schizophrenia, Schizophrenia Research, 60 : 261

Loas G., Berner P., ReinW., Yon V., Boyer P., Lecrubier Y., Traduction française du questionnaire de plaintes de Francfort (QPF) (Frankfurter Beschwerde-Fragebogen, FBF, Süllwold,1986) . L'Encéphale 1997; 23: 364 –74.

Louwerse ES, Weverling GJ, Bossuyt PM, Meyjes FE, de Jong JM (1995) Randomized, double-blind, controlled trial of acetylcysteine in amyotrophic laterl sclerosis. Archives of Neurology, Jun; 52(6):559- 64.

Terpstra M, Henry P-G, Gruetter R (in press) Measurement of reduced glutathione (GSH) in

human brain using LCmodel: Analysis of difference edited spectra. Magnetic Resonance in Medicine.

Trabesinger AH, Weber OM, Duc CO, Boesiger P (1999) Detection of Glutathione in the human brain in vivo by means of double quantum coherence filtering. Magnetic Resonance in Medicine 42: 283-289.

Yao J. K., Ravinder D. R., van Kammen P. (2001) Oxidative damage and schizophrenia: an overview of the evidence and its therapeutic implications, CNS Drugs; 15 (4): 287- 310.

Kim Q. Do Cuénod, Dr Sci Nat, PD
Cheffe de Section de Recherche
Responsable du projet
